# Engineering with keratin: A functional material and a source of bioinspiration

**DOI:** 10.1016/j.isci.2021.102798

**Published:** 2021-06-29

**Authors:** Benjamin S. Lazarus, Charul Chadha, Audrey Velasco-Hogan, Josiane D.V. Barbosa, Iwona Jasiuk, Marc A. Meyers

**Affiliations:** 1 Materials Science and Engineering Program, University of California San Diego, 9500 Gilman Drive, La Jolla, CA, USA; 2 Department of Mechanical Science and Engineering, University of Illinois Urbana-Champaign, Champaign, IL, USA; 3 Department of Materials, University Center SENAI CIMATEC, Salvador, Brazil; 4 Department of Mechanical and Aerospace Engineering, University of California San Diego, San Diego, CA, USA; 5 Department of Nanoengineering, University of California San Diego, San Diego, CA, USA

**Keywords:** Biomaterials, Engineering materials, Materials design

## Abstract

Keratin is a highly multifunctional biopolymer serving various roles in nature due to its diverse material properties, wide spectrum of structural designs, and impressive performance. Keratin-based materials are mechanically robust, thermally insulating, lightweight, capable of undergoing reversible adhesion through van der Waals forces, and exhibit structural coloration and hydrophobic surfaces. Thus, they have become templates for bioinspired designs and have even been applied as a functional material for biomedical applications and environmentally sustainable fiber-reinforced composites. This review aims to highlight keratin's remarkable capabilities as a biological component, a source of design inspiration, and an engineering material. We conclude with future directions for the exploration of keratinous materials.

## Graphical abstract

**Figure:**
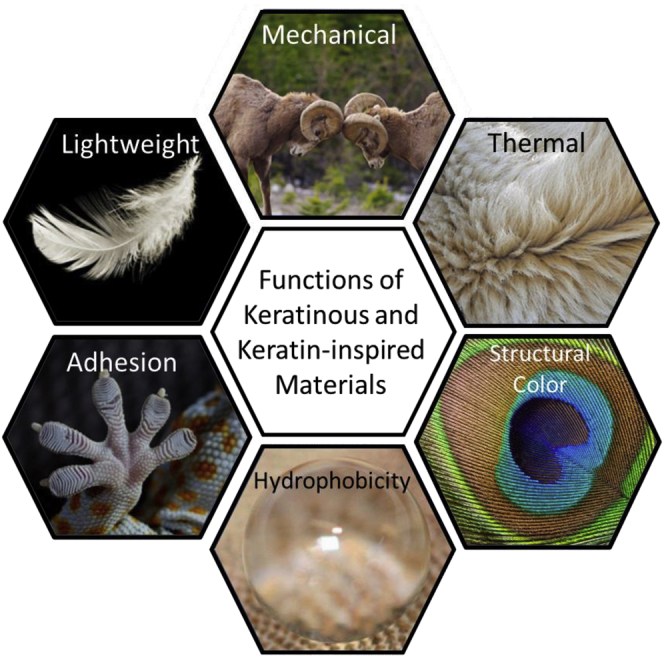

## Introduction

Keratin is a ubiquitous biological polymer comprising the bulk of mammalian, avian, and reptilian epidermal appendages, including nails, hair, the outer layer of skin, feathers, beaks, horns, hooves, whale baleen, claws, scales, hagfish slime, and gecko pads (Fraser et al., 1972; McKittrick et al., 2012; Meyers et al., 2008; Wang et al., 2016a). The omnipresence of keratin-based materials in biological systems leads to a broad range of characteristics and functions, from the impact resistance of hooves and horns to lightweight yet stiff feathers that resist buckling under aerodynamic loads. Other examples include krill filtering by whale baleen, the protective scales of a pangolin, and even the reversible dry adhesive mechanism in gecko setae that allows these lizards to climb smooth vertical walls (Huang, 2018; Labonte et al., 2016; Meyers et al., 2013). The ability of keratinous materials to perform diverse functions is derived from their ingenious structuring and tunability across many length scales. The broad array of architectures and their corresponding functions have led to the development of several keratin-inspired structures with tailored properties. Thus, keratin's structural diversity serves as a design template for the next generation of engineered materials.

Additionally, keratin has desirable intrinsic properties (biocompatibility, response to hydration, stiffness, strength, and other attributes). As a readily available and renewable material, it has been utilized as a raw material in fiber-reinforced composites. One aspect of keratin that deserves note is that it is comprised of keratinocytes after they undergo apoptosis and consists, for the most part, of ‘dead’ structures. Therefore, keratinous materials do not have the self-healing capability of living tissues such as bone. In bone, cells embedded in the structure tackle damage by repairing the torn or broken tissue.

Herein, we aim to concatenate keratin's performance as a multifunctional biological material, its use in the development of bioinspired structures, and its utilization in engineered systems. This review is organized as follows. The rest of the introduction summarizes keratin's general structure and properties as a basis for understanding the diversity of keratin-based systems. The next section, entitled “Bioinspired Materials Based on Keratinous Systems” highlights how the various structures found in keratin-based materials guide bioinspired designs across a broad range of functions (mechanical, lightweight, reversible adhesion, thermal, structural colors, and hydrophobicity). The third section, “Keratin as a Material for Engineered Systems” discusses how keratin's intrinsic material properties are harnessed for various engineering applications, focusing on biomaterials and fiber-reinforced composites. Although there are nearly twenty existing reviews of keratin and keratin-based materials, many of them focus on its structure and properties (Bradbury, 1973; Chapman, 1969a; Marshall et al., 1991; McKittrick et al., 2012; Norlén, 2006; Wang et al., 2016a; Wang and Sullivan, 2017), use as a biomaterial for biomedical applications (Donato and Mija, 2019; Feroz et al., 2020; Rouse and Van Dyke, 2010; Shavandi et al., 2017), or extraction techniques (Chilakamarry et al., 2021; Donato and Mija, 2019; Feroz et al., 2020; Khosa and Ullah, 2013; Shavandi and Ali, 2019), and there are only a few reviews that acknowledge keratin-based bioinspired materials (McKittrick et al., 2012; Wang et al., 2016a) (Table 1). None of the reviews that include bioinspiration are recent; much progress has been accomplished that warrants an updated review. This timely review illustrates how keratin obtains its vast range of functionalities from its structure and intrinsic properties and how these features are used to develop bioinspired and engineered materials. We conclude with recommendations on the future directions for keratin applications and bioinspired designs.

**Table 1:** Summary of keratin review papers

Title of review	Scope	Year	Citation
Extraction and application of keratin from natural resources: a review	Keratin structure, extraction techniques, and applications	2021	(Chilakamarry et al., 2021)
Keratin - based materials for biomedical applications	Keratin structure, extraction methods, and biomedical applications	2020	(Feroz et al., 2020)
Keratin associations with synthetic, biosynthetic and natural polymers: an extensive review	Keratin structure, chemistry, extraction methods, use as a biomaterial, and comparison to other polymers	2020	(Donato and Mija, 2019)
Keratin based thermoplastic biocomposites: a review	Techniques for using keratin to fabricate composites	2019	(Shavandi and Ali, 2019)
Keratin: dissolution, extraction and biomedical application	Keratin extraction techniques and use as a biomaterial	2017	(Shavandi et al., 2017)
A review of terrestrial, aerial and aquatic keratins: the structure and mechanical properties of pangolin scales, feather shafts and baleen plates	Keratin structure and mechanical properties	2017	(Wang and Sullivan, 2017)
Keratin: structure, mechanical properties, occurrence in biological organisms, and efforts at bioinspiration	Keratin structure, biochemistry, mechanical properties, and bioinspiration efforts	2016	(Wang et al., 2016a)
A Sustainable role of keratin biopolymer in green chemistry: a review	Keratin structure, functional properties, and chemistry	2013	(Khosa and Ullah, 2013)
The structure, functions, and mechanical properties of keratin	Keratin structure, function, and bioinspiration	2012	(McKittrick et al., 2012)
A review of keratin-based biomaterials for biomedical applications	Keratin biology, history of research, and use as a biomaterial	2010	(Feroz et al., 2020)
Stratum corneum keratin structure, function and formation – a comprehensive review	Keratin from the stratum corneum, structure, mechanical properties and modeling	2006	(Norlén, 2006)
Structure and biochemistry of mammalian hard keratin	Structure and biological formation of α-keratins	1991	(Marshall et al., 1991)
The structure and chemistry of keratin fibers	Structure and chemistry of keratin	1973	(Bradbury, 1973)
A review of the mechanical properties of keratin fibers	Structure and mechanical properties of keratin	1969	(Chapman, 1969a, 1969b)

### Structure of keratin

The term keratin originates from the Greek word ‘kera,’ which means horn. Historically, keratin denoted proteins extracted from modifications of skin such as horns, claws, and hooves. However, with an increased understanding of its structural and chemical characteristics, keratin now refers to all intermediate filament (IF)-forming proteins with specific physicochemical properties that are produced in any vertebrate epithelium (Bragulla and Homberger, 2009). These proteins form the bulk of cytoplasmic epithelial and epidermal appendageal structures (i.e., hair, wool, horns, hooves, and nails) (Wang et al., 2016a). They are also present inside cells as IFs, which provide structural stiffness, together with actin fibers and microtubules; we will not include this form in this review. This review will use the term “keratin” to describe this material at the nanoscale (macrofibrils) and below. In contrast, “keratinous material” will be used to describe the larger scale structures that are composed of these keratin fibers.

**Figure 1:**
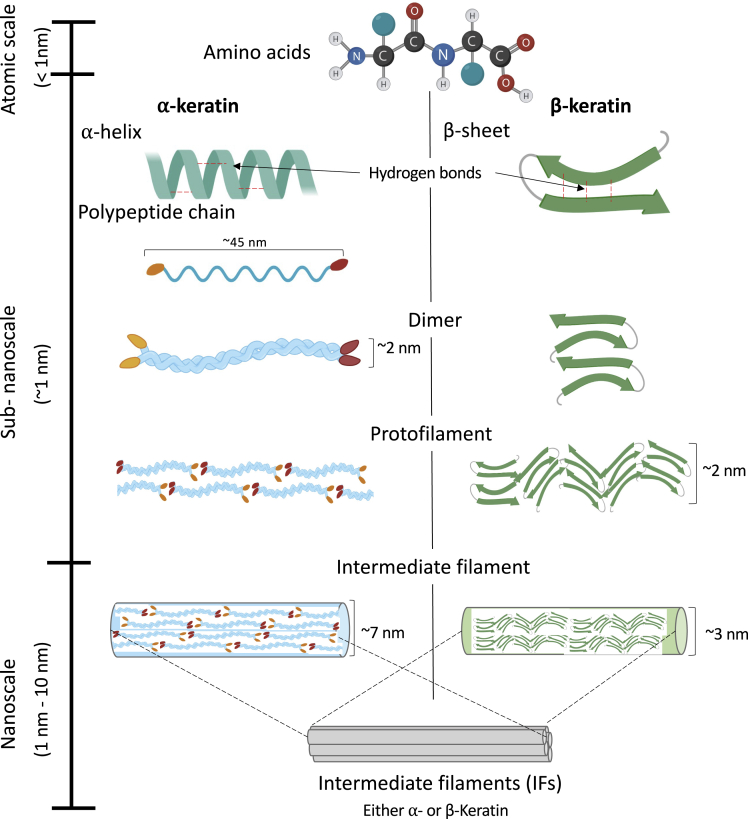
Comparison between the atomic scale and sub-nanoscale of α- and β-keratin Both α- and β-keratin, composed of amino acids, are similar at the atomic scale. The secondary protein structures are distinct for α (helix)- and β (sheet)-keratin at the sub-nanoscale. The subsequent polypeptide chains both form dimers which assemble into protofilaments and finally intermediate filaments. At the scale of IFs, both structures converge despite the differences in their diameters.

**Figure 2:**
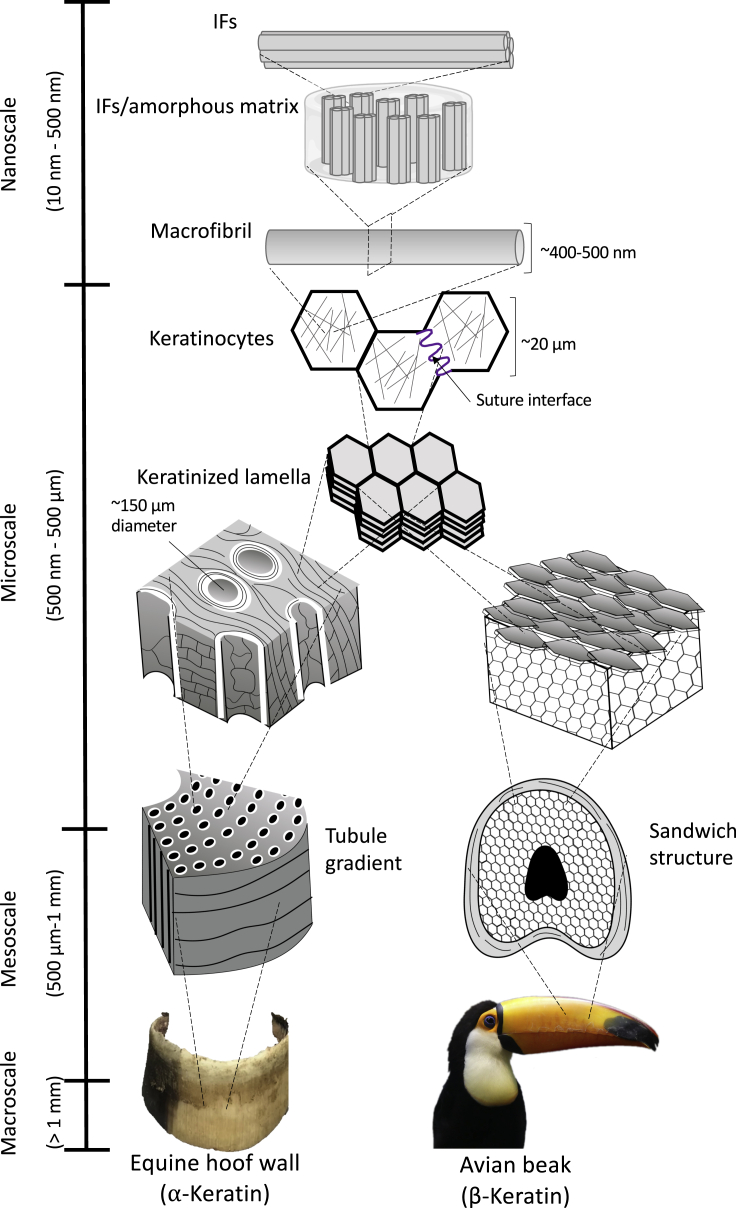
Once α and β keratin form IFs, their general structure converges before splitting at larger length scales The IFs embed in an amorphous matrix which then forms macrofibrils. These macrofibrils fill dead pancake-shaped keratinocyte cells, which stack on top of each other forming lamellae. From there, the structure of each keratinous system diverges to fulfill its specific function better. On the micro-, meso-, and macroscale, a vast range of designs and configurations are formed from the keratinous building blocks.

Keratins are broadly classified as having either α- or β-ultrastructures (Figure 1). Typically, mammalian keratin is found in the α-keratin form, while avian and reptilian keratins are β-keratin types; however, one mammal, the pangolin, is known to have both α- and β-keratin domains in its scales (Wang et al., 2016c). Like all biological materials, both α- and β-keratinous materials form hierarchical structures with geometries ranging from the atomic scale to the macroscale, as shown in Figures 1 and 2. Both α- and β-keratin are built from amino acids at the atomic level. In α-keratin, the amino acids form a right-handed α-helix secondary protein structure stabilized by hydrogen bonds (Burkhard et al., 2001; Fraser et al., 1976; Pace and Scholtz, 1998; Rojas-Martínez et al., 2020; Singamneni et al., 2019). These protein structures, also referred to as polypeptide chains, are approximately 45 nm in length and form the basic building block of an IF at the sub-nanoscale. Two polypeptide chains twist together in a left-handed rotation to form a dimer, referred to as coiled-coil (Crick, 1952). The dimers are also approximately 45 nm in length and have a diameter of ∼2 nm. It is believed that the coiled-coil structure increases the stability of the filament compared to a single α-helix (Chou and Buehler, 2012). Terminal segments of the dimer constitute an amorphous head and a tail domain. Both the head and tail regions aid in the dimer's self-assembly. The two coiled-coil dimers then aggregate together to form a tetramer which bonds lengthwise (with disulfide bonds) to create protofilaments. Two protofilaments align to form a protofibril. Four protofibrils then connect to create an IF (Bray et al., 2015). The IFs, which are ∼7 nm in diameter for α-keratin, are crystalline and are embedded in an amorphous keratin matrix. Crystalline IFs and the amorphous matrix form IF-matrix composites, which act as a basic structure for macrofibrils (∼400-500 nm in diameter). In literature, keratins are often considered short fiber-reinforced biopolymers consisting of an amorphous matrix and crystalline fibers (IFs)(McKittrick et al., 2012).

Similar to α-keratin, β-keratin is composed of amino acids at the atomic scale and has a comparable hierarchical order (dimer to protofilament to IF) at the sub-nanoscale. The most significant difference, compared with α-keratin, is that β-keratin has a different secondary protein structure characterized by pleated β-sheets (Toni et al., 2007). In β-keratins, the antiparallel peptide chains are positioned side-by-side to form a rigid planar surface. These surfaces are slightly bent with respect to each other, creating a pleated arrangement (McKittrick et al., 2012). The planarity of the peptide bond and the lateral hydrogen bonding accounts for the formation of the pleated sheet (Fraser and Parry, 2011). Similarly to α-keratin, the β-sheet self-assembles into a dimer, which forms the basis of the distorted β-sheet (called a protofilament). Protofilaments align to form the β-keratin IF, which is ∼3 nm in diameter. For β-keratin, the terminal sections of the polypeptide proteins wrap around the filaments to form the amorphous matrix. Besides the differences between the α- and β-keratin at the sub-nanoscale, both keratin types form similar hierarchical structures up to the nanoscale (Figure 2). At the microscale, keratinous materials' architecture diverges for different organisms to optimize their structures for their specialized functions.

At the nanoscale, the IFs are embedded in an amorphous matrix in both α- and β-keratins. This IF-matrix nanocomposite structure subsequently groups to form macrofibrils (∼400-500 nm in diameter) and then fibers (∼6 μm). Variations in the IF alignment, volume fraction, orientation, and matrix properties account for the wide range of mechanical properties of keratin-based structures. Keratinocytes are the once living cells that are filled with keratin fibers. Their formative boundaries encapsulate the orientation and can vary across organisms or locations within a specific organism. When stacked together, the keratinocytes form a layered structure at the microscale due to their inherent directional growth from the follicle. In some systems such as the horse hoof wall, woodpecker beak, pangolin scale, and bighorn sheep horn, the interface between neighboring keratin cells exhibits a wavy sutured morphology. Through their layered growth, keratin cells form laminated sheets. The hierarchical structure of many keratinous systems begins to diverge at this scale. This layered structure is a defining feature of keratin-based materials. The laminated sheets organize themselves into different arrangements at the mesoscale. For example, the laminated structure in some horns and hooves is characterized by embedded microtubules, whereas the lamellae in hair cuticles have an overlapping configuration. Even more so, at these larger length scales, some keratinous materials begin forming cellular solids such as the foamy centers of quills and feather shafts. The divergence of the structure at the meso and macroscales for each organism will be explained in greater detail in Section 2.

**Figure 3:**
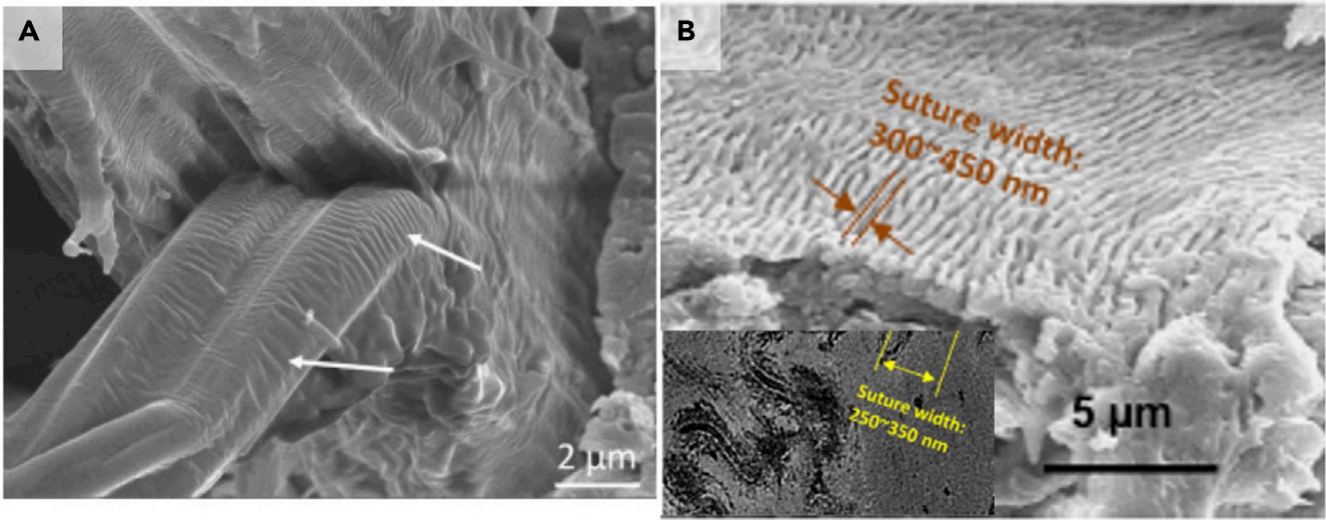
Intercellular suture structures are present on the surface of many keratinocytes (A) Human hair. Reproduced with permission (Yu et al., 2017a). Copyright 2017, Elsevier. (B) Pangolin scales. Reproduced with permission (Wang et al., 2016c). Copyright 2016, Elsevier.

There are also morphological differences among different keratinocytes: in hair, they are elongated along the axis (one dimension much larger than the other two); in pangolin scales and many other places, they are pancake-shaped, with one dimension much smaller than the other two. There seems to be a preponderance of suture structures at the mesoscale. The surface of a cortical cell in human hair after tension exhibits a suture-like structure, which increases the contact area of cortical cells and therefore increases the adhesion between adjacent cells and decreases splitting of hair along the axis. This suture structure is also found in the pangolin scale. It has a width between 250 and 450 nm and creates an interlocking effect. This structure has been studied and generalized by the Ortiz group (Li et al, 2011, 2012, 2013; Lin et al., 2014a, 2014b). Figure 3 shows the suture structures in hair and pangolin scale.

To fully capture the hierarchical structure of keratin, computational models have been developed for each length scale. Starting with the fundamental building blocks of amino acids, these models aim to analyze the mechanical properties and arrangement of molecules in IFs at the sub-nanoscale (Chou and Buehler, 2012; Qin et al, 2009, 2011; Qin and Buehler, 2011). Chou and Buehler (2012) pioneered this effort by reconstructing heterodimers from an entire amino acid sequence of keratin proteins using molecular dynamics simulations. The geometric dimensions of the reconstructed dimer matched well with the experimental observations. Using this model, they compared keratin's mechanical properties with and without disulfide bonds and concluded that the disulfide bonds improve keratin's durability and strength (Chou and Buehler, 2012). This feature is similar to the one in elastomers, where vulcanization introduces sulfur bonds between the chains and increases the performance markedly. Qin et al. (2009), from the same Buehler group, discussed the hierarchical structure of IFs and analyzed each hierarchical level's influence on the IF's mechanical properties. They divided the hierarchical structure of IFs at the atomic scale and sub-nanoscale (Figure 1) into additional eight hierarchical levels. Using molecular dynamics modeling, they concluded that each hierarchical level demonstrates a distinct deformation mechanism, which enables keratin to sustain prominent deformation at higher length scales (beyond the nanoscale) (Qin et al., 2009). The dominant mechanisms at each hierarchical level and their description are summarized in Table 2. In another paper, Chou et al. (2015) demonstrated how information from atomic-scale models could be utilized to predict human hair's mechanical properties at the mesoscopic scale through a bottom-up approach (Chou et al., 2015).

**Table 2:** Key hierarchical levels and their corresponding mechanisms

Length scale	Hierarchical structure	Key mechanism
Atomic scale	Amino acid ordering and hydrogen bonding	Hydrogen bonding forms at moderate temperatures and prompts formation of alpha-helices
Sub-nanoscale	Alpha-helix and Beta-sheet	Alpha-helical turns permit large tensile strains and extensibility due to uncoiling.
Sub-nanoscale	Dimer	Increased stability and resistance to mechanical deformation
Sub-nanoscale	Protofilament	Increased resistance to interfilament shear
Sub-nanoscale	Intermediate filament	Increased extensibility, stiffening, and superplastic properties
Nanoscale	IFs embedded in amorphous matrix	IFs provide rigidity while the amorphous matrix distributes the applied load
Nanoscale	Macrofibril	Increased rigidity and extensibility
Sub-microscale	Keratinocytes	Organization of macrofibrils by cell boundaries
Sub-microscale	Suture interface	Provides interlocking interface between neighboring cells, enhances flexibility, and tailored stiffness.
Microscale	Keratinized lamella	Layered structure makes up the relative thickness of the material and distributes stress across the material. Allows for local flexibility and increases extensibility due to sliding of lamella.
Mesoscale	Dependent on material but can include tubules, sandwich structures, etc.	Dependent on structure. Tubules provide compressibility and crack deflection. Sandwich structures are lightweight yet stiff.

### Mechanical properties of keratin

The polymeric nature of keratin lends itself to a wide range of mechanical properties that vary according to its amino acid composition, structure, and hydration level (Bertram and Gosline, 1987; Fraser and Parry, 2014; Greenberg and Fudge, 2013; McKittrick et al., 2012; Wang et al., 2016a). The amino acid sequence and corresponding residues dictate the availability of disulfide bridges. The amino acid cysteine has a thiol group which allows for a covalently bonded di-sulfide bond to be formed with another cysteine further along the chain and creates a fold in the protein. Chou and Buehler (2012) showed that keratin's hardness is strongly correlated with the density of sulfur cross-links (Chou and Buehler, 2012). A low amount of sulfur indicates soft keratins (outer layer of skin, i.e., stratum corneum). In contrast, a high amount of sulfur leads to hard keratins (e.g., hair, nails, feathers, hooves) (Chou and Buehler, 2012; Parbhu et al., 1999; Smack et al., 1994).

**Figure 4:**
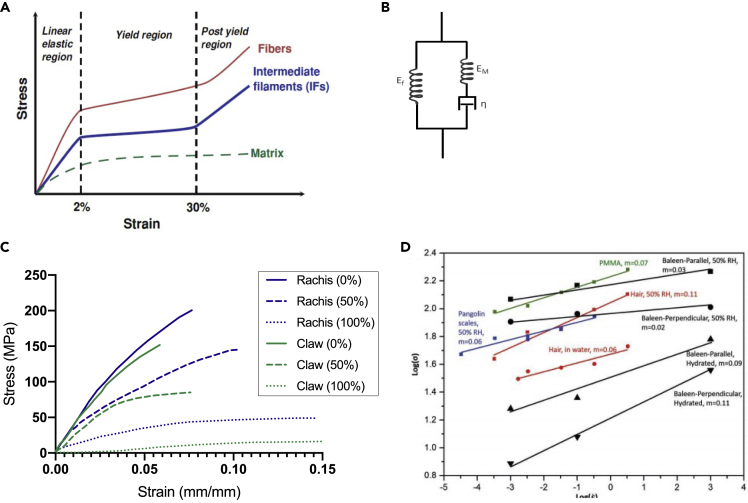
Mechanical properties of keratin and keratinous materials (A) Idealized stress-strain curve of α-keratin showing three distinct regions. This is a representative curve and does not take into account factors like viscoelasticity or structural deformation mechanisms. Still, it does highlight the plateau yield region and the range of these three phases of deformation. Reproduced with permission (McKittrick et al., 2012). Copyright 2012, Springer. (B) Spring and dashpot configuration of the two-phase model that is used to incorporate the hydration-induced viscoelasticity of the amorphous matrix. (C) Tensile stress-strain curves of bird feathers and claws test at different humidities at a strain rate of 0.11 min^−1^. Adapted with permission (Taylor et al., 2004). Copyright 2004, Springer. (D) Effect of strain rate on biopolymers' strength (whale baleen, hair, pangolin) and the synthetic polymer PMMA. Reproduced with permission (Wang et al., 2019). Copyright 2018, Wiley.

Based on the structural arrangement described in the previous section, keratin's amino acid chains can either curl into helices (α-configuration) or bond side-by-side into pleated sheets (β-configuration). The molecular arrangement associated with the alignment of IFs directly influences the mechanical properties of keratinous materials (McKittrick et al., 2012). The stress-strain curve of a typical α-keratinous material consists of three distinct regions: linear elastic region, yield region, and post-yield region, as shown in Figure 4A. Figure 4A decomposes the contributions of both the IFs and the matrix to the properties of α-keratin fibers. The linear elastic region extends approximately up to a 2% strain. In this region, the stress increases linearly with an increase in strain (Chapman, 1969b). Beyond 2% strain, the keratinous material enters the yield region in which it reaches critical stress beyond which the coiled-coil region of the α-keratin helices begins to unravel into the β-pleated sheet structure exhibited by β-keratin (Cao, 2002; Kreplak et al., 2004; Paquin and Colomban, 2007). As a result, the stress-strain curve exhibits a large plateau. X-ray diffraction studies have shown that microfibrils open at various points and increase in length during the conversion (Bendit, 1957). However, atomic-scale simulations have demonstrated that the structure of the dimer assembles in a specific sequence (Chou and Buehler, 2012). The low increment in stress in the yield region can be explained by the Ciferri model (Ciferri, 1963). Ciferri proposed that the low increment in stress is due to thermodynamic equilibrium existing between α-and β-structures. The α- and β-keratins coexist in equilibrium at a constant stress value dependent on temperature but not on each state's relative quantities. The plateau region exists up to ∼30% strain, beyond which the material enters the post-yield region, where the stress again increases with an increase in strain. The rise in stress can be attributed to the coupling between the matrix and IFs. Even though the α-keratin continues to convert to β-keratin until 70-80% of strain, the matrix starts resisting deformation at ∼30% strain and thus begins to bear additional stress. As a result, a sharp rise in tangent modulus is observed (Ciferri, 1963).

Several attempts have been made to capture the mechanical properties of keratin analytically. The most notable ones are the two-phase model proposed by Feughelman (Feughelman, 1959) and the Hearle-Chapman model (Chapman, 1969b; Hearle, 1967). The initial two-phase model of Feughelman was later modified to incorporate additional features of keratin. In this revised model, the keratinous material comprises two phases: C and M. Phase C denotes long water-impenetrable and relatively rigid cylindrical rods. These rods are embedded in a water-absorbing matrix called phase M. Phase C represents a coiled-coil part of the polypeptide chain in α-keratin. This phase has lower sulfur content to interact with water. Phase M consists of non-helical parts of α-keratin (like its head and tail) and matrix structure surrounding polypeptide chains. These parts have higher sulfur content and can absorb water, giving rise to viscoelastic behavior in keratin. According to this model, the initial region (named the linear elastic region by earlier, less complex studies) of the stress-strain curve for α-keratin can be represented by a spring and dash-pot model (Figure 4B) where a spring (with a spring constant of E_f_) is in parallel with another spring (with a spring constant, E_M_) and dashpot (with viscosity, η). The spring constant, E_f_, represents Young's modulus of the crystalline phase and therefore does not depend on moisture content. The E_M_ and η represent the properties of a viscoelastic amorphous matrix dependent on moisture and temperature. As evident from the spring-dashpot model, the non-linear viscoelastic behavior of keratin in the Hookean region is due to the matrix phase described as a weak “gel” structure (Feughelman, 2002). As the gel structure is extended at a fixed rate, the bonds progressively break down. If the extension is ceased, the broken bonds re-form rapidly in equilibrium.

In the yield region, the α-helices in the crystalline phase C are extended to the fiber structure's total length. As a result, they start unfolding to β-units at a nearly constant stress, governed by a thermodynamic equilibrium between α- and β-units. Most of the force applied to the keratinous material in this region is resisted by the IFs, whereas phase M resists only a small force that is nearly constant. The viscosity contributes to the time constant for the relaxation and provides resistance to folding and unfolding of α-helices.

**Figure 5:**
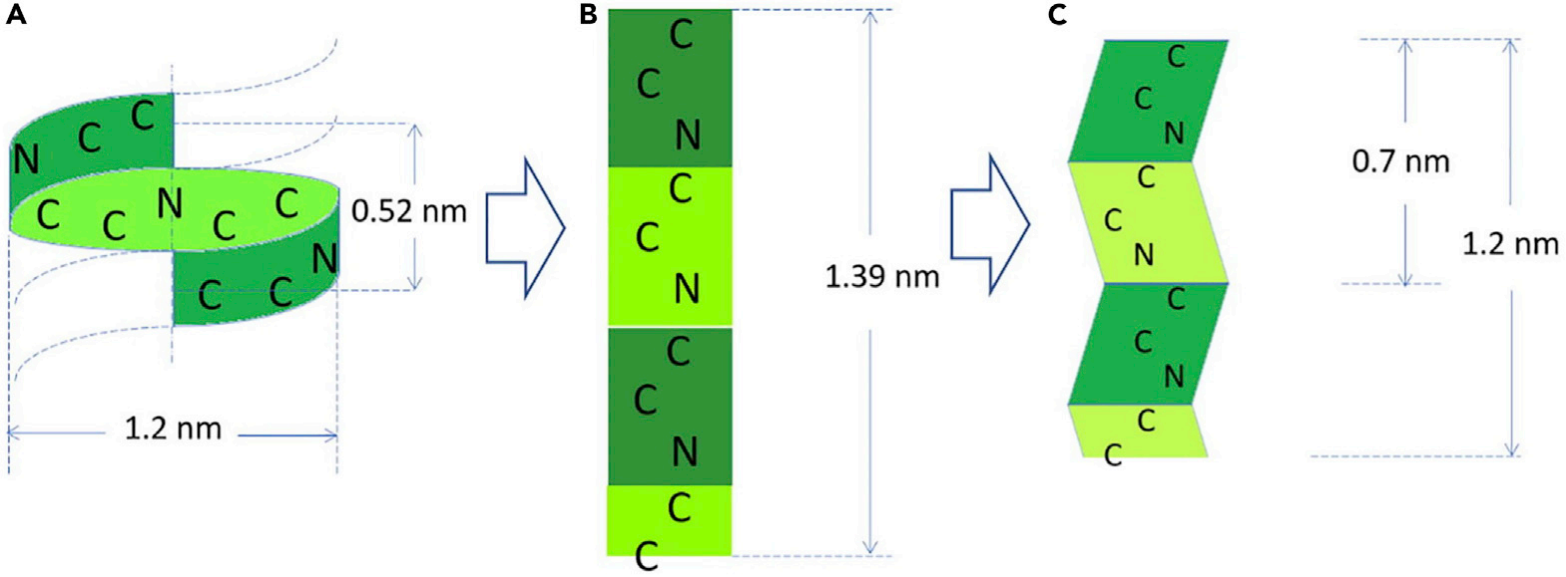
Full period (one rotation, corresponding to -CCNCCNCCNCC-) for α-helix (0.52 nm) and corresponding distance for β-pleated sheet (1.2 nm) The stretched β configuration with the same chain (-CCNCCNCCNCC-) has a length of 1.39 nm. The formation of pleats reduces the length to 1.2 nm. The theoretical strain corresponding to full transformation is equal to 1.34; this is seldom achieved in real cases. Reproduced with permission (Yu et al., 2017a). Copyright 2017, Elsevier.

When the α-helices transition to β-pleated sheets in the yield region, they extend in length. Figure 5A shows a full period of the α-helical structure consisting of the atomic sequence (*-CCNCCNCCNCC-)*; its length is 0.52 nm. When this helix is fully rectified and extended (Figure 5B), its length becomes 1.39 nm. However, the assembly of polypeptides is such that a folded β-pleated sheet is formed; this reduces the length to 1.2 nm. Thus, the nominal strain of the α to β be calculated and is equal to 1.34. However, it is rarely achieved experimentally, and other processes are thought to take place.

At a larger spatial scale, the IFs parallel to each other start moving closer together, jamming the still unfolding α-helices against the matrix phase, which consists of globular matrix proteins. Owing to the increase in length when the α-helices transition to β-pleated and the jamming of proteins in the matrix, further extension of the material distorts matrix proteins. As a result, the matrix starts carrying more load resulting in an increase in stress with strain. The above is the essence of the Feughelman model.

Chapman (Chapman, 1969b) and Hearle et al. (Hearle, 1967) independently extended the two-phase model to explain the zonal unfolding of α-helices in microfibrils by considering the effect of mechanical coupling between the fibril and matrix. They assumed that the single fibril is of infinite length. The matrix never enters the yield region and therefore behaves elastically as it bears only a small portion of the total force. Based on this model, they derived the equations to predict stresses and strains in different regions as shown below. We use subscripts f and M for the fiber and matrix, respectively.

Hookean region: $E_{M}\ll E_{f}$ thus, the stress is taken by the fibril

$$\sigma =\;E_{f}\varepsilon$$

At the yield point

σ=σcandε=εc=σcEf

Yield region: (once the transition of α-fibrils has started)

ε=σEf+ε2

$$\sigma_{c}=\;\sigma_{e}+E_{M}\varepsilon_{M}$$

End of post yield region

$$\sigma_{0}=\;\sigma_{e}+E_{M}\varepsilon_{b}$$

ε0=σEf+εb

where

$$\frac{1}{E_{p}}=\;\frac{1}{E_{f}}+\;\frac{1}{E_{M}-\left(\sigma_{c}-\sigma_{e}\right)/\left(2\varepsilon_{b}\right)}$$

$E_{M}$ is Young's modulus of the matrix, $E_{f}$ is an initial fibril modulus,σ and ε are the total stress and strain, respectively, in the material.$\;\sigma_{c}$ is equal to the critical stress at which the unfolding of α fibrils begins. $\varepsilon_{2}$ is the strain due to unfolding of α fibrils,$\;\sigma_{e}$ is the equilibrium stress for the transition between α to β fibrils, $\varepsilon_{M}$ is the strain in the matrix, $\varepsilon_{b}$ is the strain associated with α to β transition, and $E_{p}$ is the effective modulus in a post-yield region. The detailed derivation for the above equations is given in Hearle and Chapman (Chapman, 1969b; Hearle, 1967).

In general, α-keratin has a high tensile fracture strain, primarily due to the stretching and sliding of the polymer chains across many length scales. The hagfish slime threads have the highest tensile breaking strain of 2.2 when tested in seawater (Fudge et al., 2003). Despite large tensile breaking strains, there are significant variations in tensile strength across species due to structural orientation, hydration, and composition (Fudge et al., 2003). The tensile strength ranges from 2 MPa in the stratum corneum to 225 MPa in human hair to 530 MPa in the hagfish's dry slime threads. Mechanical properties of keratinous materials also depend on the orientation and volume fraction of IFs: greater alignment of the IFs results in a higher tensile strength along the alignment direction. Thus, the tensile strength of human hair (where all the IFs in the cortex are aligned with the hair axis) is higher than that of human nails (where there are three layers in which the IFs are oriented at 90° to each other).

The degree of hydration dramatically influences the mechanical properties of keratin. Increasing humidity and water content decreases the stiffness, strength, and hardness (Collins et al., 1998; Johnson et al., 2017; Liu et al., 2016a; Wang et al., 2016a) This behavior, summarized in Table 3, is attributed to the interaction of water molecules with the amorphous matrix, which breaks stabilizing hydrogen bonds and increases the mobility of the fibers within the matrix (Winegard and Fudge, 2010). In equine hoofs, Young's modulus drops an order of magnitude between dry and hydrated conditions (Bertram and Gosline, 1987; Kasapi and Gosline, 1999). This increase in ductility in hydrated keratinous samples is associated with a higher tensile strain but lower tensile stress. Thus, hydration has a drastic effect on strength. The feather, for example, sees its tensile strength more than halved from 221 MPa to just 106 MPa when placed in 0% relative humidity (RH) environment vs. 100% RH environment (Taylor et al., 2004). These trends can be seen in Figure 4C, which shows the stress-strain curves of bird feathers (rachis) and claws under tension at different relative humidities. Additionally, the pangolin scale has been shown to exhibit a decrease in hardness with hydration, from 314 MPa to 148 MPa in dry and hydrated states, respectively (Liu et al., 2016a). Other systems like whale baleen, porcupine quill, horn, and claws also see drastic reductions in strength with increasing hydration.

**Table 3:** Mechanical properties of keratinous systems at various humidity levels

Biological material	Humidity	Young's Modulus (GPa)	Strength (MPa)	References
Stratum corneum	10% RH 100% RH	1 0.005	18 2	(Wu et al., 2006)
Wool	0% RH 65% RH 100% RH	– 4.5 2.5	260 – 180	(Fraser and Macrae, 1980; Morton and Hearle, 2008)
Quill	65% RH 78% RH 100% RH	2.7 1.9-2.3 1.0	146 61.3–167.9 60	(Yang et al., 2013)
Horn	50% RH Soaked in water	3.9 0.7	77 25	(Trim et al., 2011)
Hoof	0% RH 75% RH 100% RH	14.6 2.63 0.41	– 38.9 9.18	(Bertram and Gosline, 1987)
Whale baleen	Ambient Soaked in water Soaked in water	1.8/3.1 (perp./para.) 0.1/1.1 (perp./para) 1.2	80/116 (perp./para.) 7/19 (perp./para.) 30	(Szewciw et al., 2010; Wang et al., 2019)
Hagfish slime threads	Soaked in water	0.006	180	(Fudge et al., 2003)
Feather	0% RH 100% RH	3.7 1.5	221.0 106.3	(Taylor et al., 2004)
Beak	50% RH	1.3	47.5	(Seki et al, 2005, 2006)
Claw	0% RH 50% RH 100% RH	2.7 2.1 0.14	90.3 68.7 14.3	(Taylor et al., 2004)
Pangolin scale	50% RH	0.963	72.43	(Wang et al., 2016c)
Snake epidermis	43% RH	3.42-4.73	–	(Klein et al., 2010)
Finger nail	0% RH 55% RH 100% RH	4.34 2.32 0.47	– – –	(Farran et al., 2009)
Hair	20% RH 50% RH Soaked in water	4.2	∼250 ∼175 ∼165	(Yu et al., 2017b)
Gecko seta	30% RH 80% RH	3.7 2.13	262.5 237	(Prowse et al., 2011)

∗% RH = % Relative humidity, perp. = perpendicular to longitudinal axis of the tubules, para. = parallel to longitudinal axis of the tubules.

There are apparent variations in the mechanical properties of different keratinous systems. For example, the hoof, which has reinforced tubules, exhibits Young's modulus of 14.6 GPa at 0% RH, more than three times that of fingernails, claws, and feathers under the same humidity conditions. Even keratinous materials found in similar organisms, such feathers and claws, have noticeably different mechanical behaviors. These variations can also be observed in Figure 4C. These differences can result from deviations in both chemical composition (i.e., mineralization, degree of crystallinity, etc.) or structure (porosity, lamellar arrangement, fiber orientation, etc.)

Keratin is known to be highly strain-rate sensitive, which is related to its viscoelasticity and viscoplasticity, i.e., its time-dependent response (Yu et al, 2017a, 2017b). This is a typical behavior of polymers. Figure 4D shows the strength (*σ*) versus strain rates ($\dot{\varepsilon }$) on a log-log scale for whale baleen, hair, pangolin scales, and a synthetic polymer (polymethyl methacrylate [PMMA]); the similarity is evident. The strain rate sensitivities “m” (defined as $\frac{d\left(log\sigma \right)}{d\left(log\dot{\varepsilon }\right)}$) for biological materials (hair, pangolin, whale baleen) are comparable to those of PMMA, a synthetic polymer. In the case of whale baleen, the strain-rate sensitivity of the dry samples (m ≈ 0.02–0.03) is significantly lower than that of the hydrated ones (m ≈ 0.09–0.11). This difference is attributed to the hydrated specimens' increased viscosity, enabled by the water molecules penetrating the amorphous matrix and plasticizing it. In the dry specimens, the effect of the mineral phase becomes stronger.

The general trend for keratinous materials is that increasing strain rate increases stiffness and strength, while decreasing the breaking strain (Johnson et al., 2017; Kasapi and Gosline, 1996; Seki et al., 2005; Song et al., 2009). Thus, most keratin materials undergo an elastic to ductile-plastic to brittle transition with an increasing strain rate, as was shown for the toucan rhamphotheca (Seki et al., 2005) and pangolin scales (Wang et al., 2016c). This rate-dependent behavior has important implications for impact resistance, suggesting that these materials can withstand greater stresses under dynamic conditions and have different failure mechanisms than quasi-static conditions. The embrittlement at high strain rates is an important consideration.

**Figure 6:**
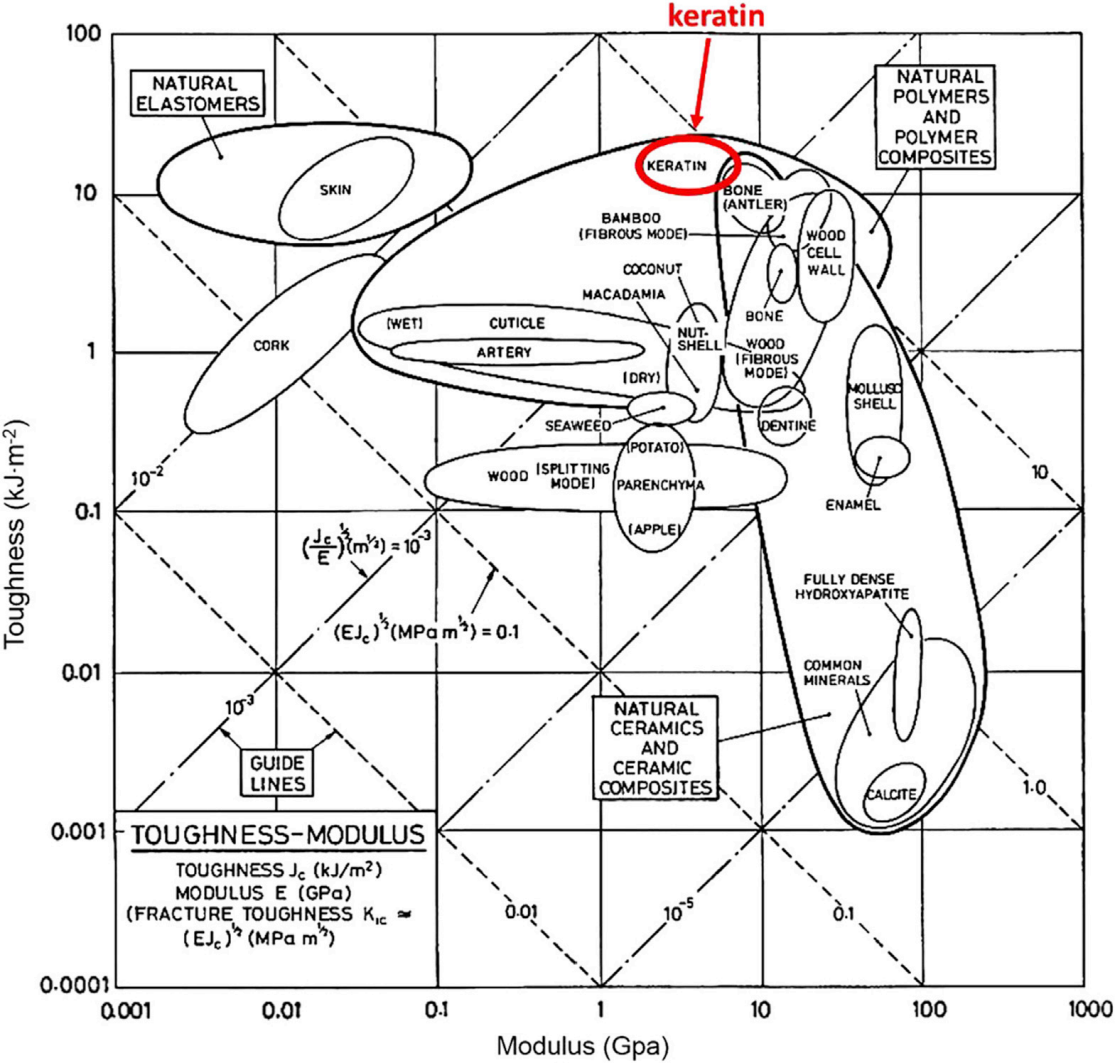
Ashby diagram demonstrating toughness vs. modulus for different biological material Reproduced with permission (Wang et al., 2016b). Copyright 2016, Elsevier.

Keratin is also one of the toughest biological materials, as seen in Figure 6 (Wang et al., 2016a). This characteristic is due primarily to its hierarchical structure. As demonstrated by Qin et al. (2009), different hierarchical levels can undergo distinct deformations that enable keratin to absorb larger amounts of energy before failure (Qin et al., 2009). The matrix is primarily responsible for distributing the applied loads during large deformations, while the fibers carry the most load and serve to arrest cracks. Some keratinous materials have optimized mesoscale features, such as tubules in horns and equine hooves, which enhance the material's toughness. Due to fiber orientation, fiber concentration, and the presence of features like tubules along a specific direction, toughness is typically found to be anisotropic (Bertram and Gosline, 1986).

### Hydration-induced shape recovery

**Figure 7:**
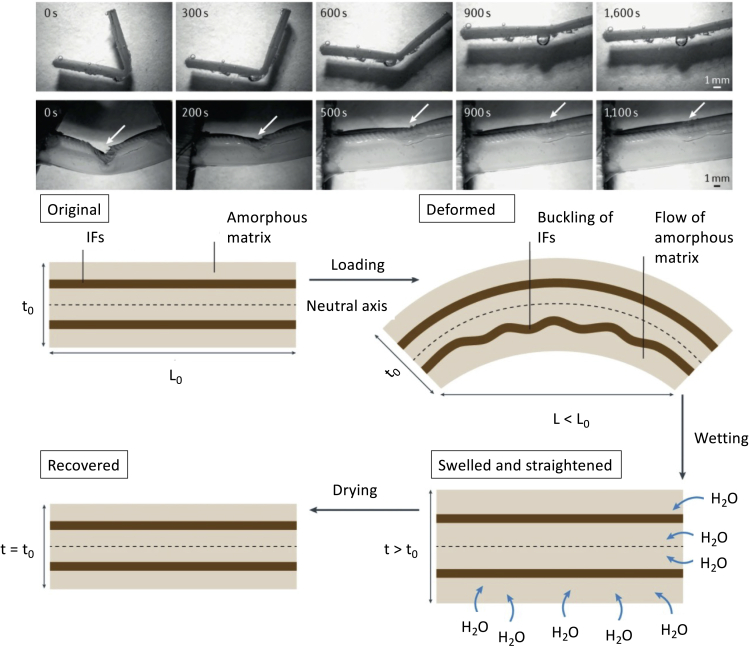
Hydration-induced mechanical reversiblilty is a common trait amongst keratinous systems Reversible deformation of the feather shaft induced by hydration; top: restraightening of a deformed feather with hydration and recovery of its initial shape; bottom: sequence of events as the IF-amorphous matrix composite is first deformed and then hydrated. Adapted with permission (Quan et al., 2021). Copyright 2021, Nature.

Keratin systems often function as protective layers which undergo significant deformation. Many of these systems are permanent and cannot remodel or self-heal through biological processes after experiencing considerable deformation, such as in the bighorn sheep horn (Huang et al., 2019b), feathers (Liu et al., 2015b; Sullivan et al., 2018), and pangolin scales (Liu et al., 2016b). A solution to this lack of regenerative capacity is keratin's ability to undergo hydration-assisted shape recovery. This phenomenon was discovered by Liu et al. (2015b), who observed 98% shape recovery in compressed peacock tail feathers after seven cycles of deformation to over 90% strain (Liu et al., 2015b). After the keratin is deformed plastically, the recovery process involves water infiltrating the amorphous keratin matrix, causing swelling, which forces the deformed crystalline regions of the IFs to regain their initial shape by breaking and reforming hydrogen bonds (Huang et al., 2019b). Also, the feather shaft was shown to have hydration-assisted shape and strength recovery. The feather shaft was subjected to bending and then allowed to soak in water for 24 hr, and after one cycle, it was found to recover its strength by ∼80% (Sullivan et al., 2018). The mechanism proposed by Sullivan et al. (2018) for the feather is shown in Figure 7 (Sullivan et al., 2018). The Bighorn sheep horn was also shown to recover its shape by soaking in water after severe compression of 50% strain which was further assisted by the hollow tubules (Huang et al., 2019b). In a similar study by Liu et al. (2016b), the pangolin scale was shown to have hydration-assisted strength recovery after indentation, which simulated penetration-induced injury by a predator. The self-healing was attributed to the swelling of the keratin-based material allowing for an increase in flexibility of keratin fibers to reorientate and straighten (Liu et al., 2016b).

The top sequence of Figure 7 shows the gradual restraightening of the feather shaft as it is hydrated. Plastic deformation causes permanent deformation of the amorphous matrix (bottom sequence), which is weaker than the IFs. The IFs undergo buckling on the compression side. Upon hydration, water molecules penetrate the amorphous matrix and cause swelling, which forces the crystalline IFs to straighten and realign. Upon drying, the matrix shrinks again, and the original configuration is established. These studies show that hydration actuates shape recovery in α-keratin and β-keratin, which is not surprising as both keratins have similar structures involving crystalline IFs embedded in an amorphous matrix.

### Thermal properties

Another common function of keratin is to serve as a thermal insulating barrier in hair, wool, fur, and feathers, to name a few. Often the goal of these systems is to trap air pockets within the insulating layer. This method is very effective since air has an extremely low thermal conductivity of just ∼0.0264 Wm^−1^K^−1^ (Mao and Russell, 2007). As noted previously, the self-assembly process of natural keratinous materials has afforded some organisms with precisely controlled meso-, micro-, and nanostructures. For thermal insulation, this ability has been utilized to generate lightweight systems that trap significant amounts of air with minimal material. Note that keratin by itself has a low thermal conductivity of just 0.19 Wm^−1^K^−1^. However, when arranged into low-density wool, the combined thermal conductivity is reduced to 0.03 W m^−1^K^−1^ (Mao and Russell, 2007). Nature's ability to produce these intricate structures in abundance has made certain keratinous systems like feathers, wool, and fur some of humanity's most valuable thermal insulators to date. In humans, bipedalism concentrates exposure from the sun to the head, and this is exactly where capillarity is highest. The remainder of the body is only covered by vestigial hair, and this enables an increase in sweat glands, which enhances the ability of the body to regulate the temperature and has helped humans to develop an amazing ability to run for extended distances.

## Bioinspired materials based on keratinous systems

Keratin is one of the most essential biopolymers found in nature, appearing in the integument of many vertebrates, as discussed in Section 1. Keratinous materials are especially intriguing due to their hierarchical structures, which vary widely across organisms and are found in a broad range of morphologies that are tuned for their specific functions. To show that these configurations give rise to the high performance of natural keratinous materials and can be a source of bioinspiration, these naturally occurring geometries are replicated in engineered materials by simplifying integral designs and scaling them to more appropriate sizes for processing and mechanical testing. Additionally, many of these studies rely on numerical and analytical models to better understand the mechanical behavior and deformation mechanisms of these bioinspired systems. This section will review these efforts through a bioinspired lens, focusing on how keratin-based systems and their structures achieve diverse functions.

**Figure 8:**
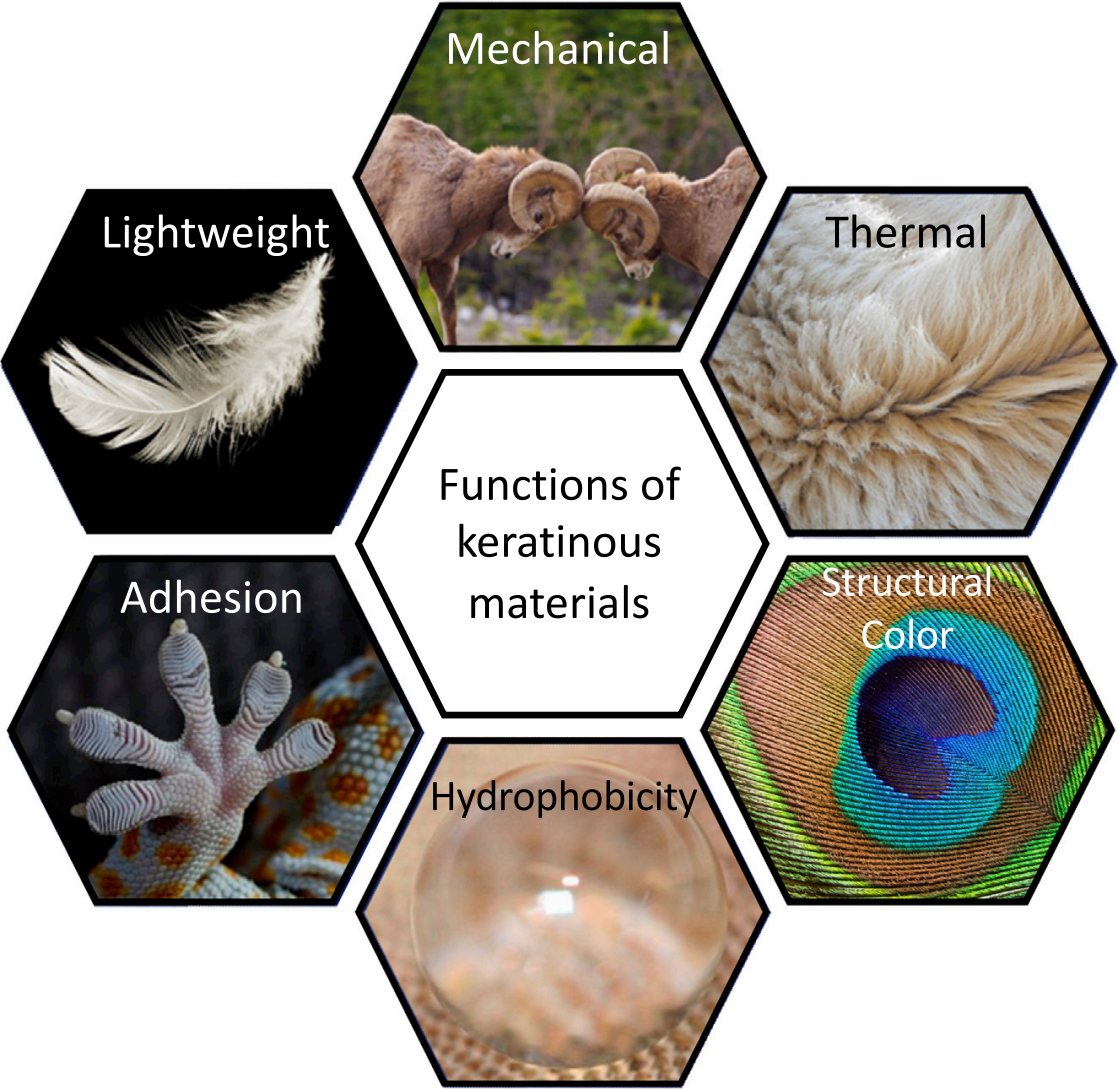
Keratin provides many functions in nature In the following section, bioinspired designs based on keratinous systems will be broken down into the classifications shown in this figure.

The many functions of keratinous materials, shown in Figure 8, will lay the framework for reviewing their associated bioinspired materials. Table 4 highlights some common examples of systems for each function and their relevant structures.

**Table 4:** Keratin biological systems, their principal functions, and related structures

Function	Biological systems	Structures	References
Mechanical	Hooves, horns, bird beak, turtle scutes, pangolin scales	Tubules, sutures, layers, sandwich structures, articulated scales	(Huang et al., 2017, 2019a, 2019b; Magwene and Socha, 2013; Wang et al., 2013, 2016c)
Lightweight	Feathers, beak, porcupine quill	Sandwich structures, foam	(Seki et al., 2010; Sullivan et al., 2017; Yang et al., 2013)
Thermal	Hair, fur, and feathers	Large surface area, trapped air	(Cui et al., 2018; Gao et al., 2007; Yu et al., 2017b)
Structural color	Feathers	Nanostructures	
Reversible adhesion	Gecko setae	Branched structures, nanospatula	(Autumn et al., 2002)
Hydrophobicity	Feathers, gecko skin	Spinules, hamuli, nanogrooves	(Liu et al., 2008; Watson et al., 2015)

### Mechanical applications

Keratin-based materials are frequently utilized in nature as structural load-bearing components that provide protection and withstand high impact forces. Keratinous systems perform admirably under such diverse mechanical demands, even compared to some of the most advanced engineered materials (Lee et al., 2011). One reason is that keratin's mechanical properties can be tuned by hydration, providing a stiff (∼10 GPa) load-bearing material when dry or a ductile rubbery material when fully hydrated (∼0.1 GPa) (Bertram and Gosline, 1987; Collins et al., 1998; Huang et al., 2019a; Zhang et al., 2007). Another reason is that keratin takes on the form of a wide range of structures with intricate geometrical features at multiple length scales that synergistically lead to high mechanical performance. This subsection will review keratinous systems with remarkable mechanical properties and instances where their structural features have been used as inspiration for synthetic materials.

**Figure 9:**
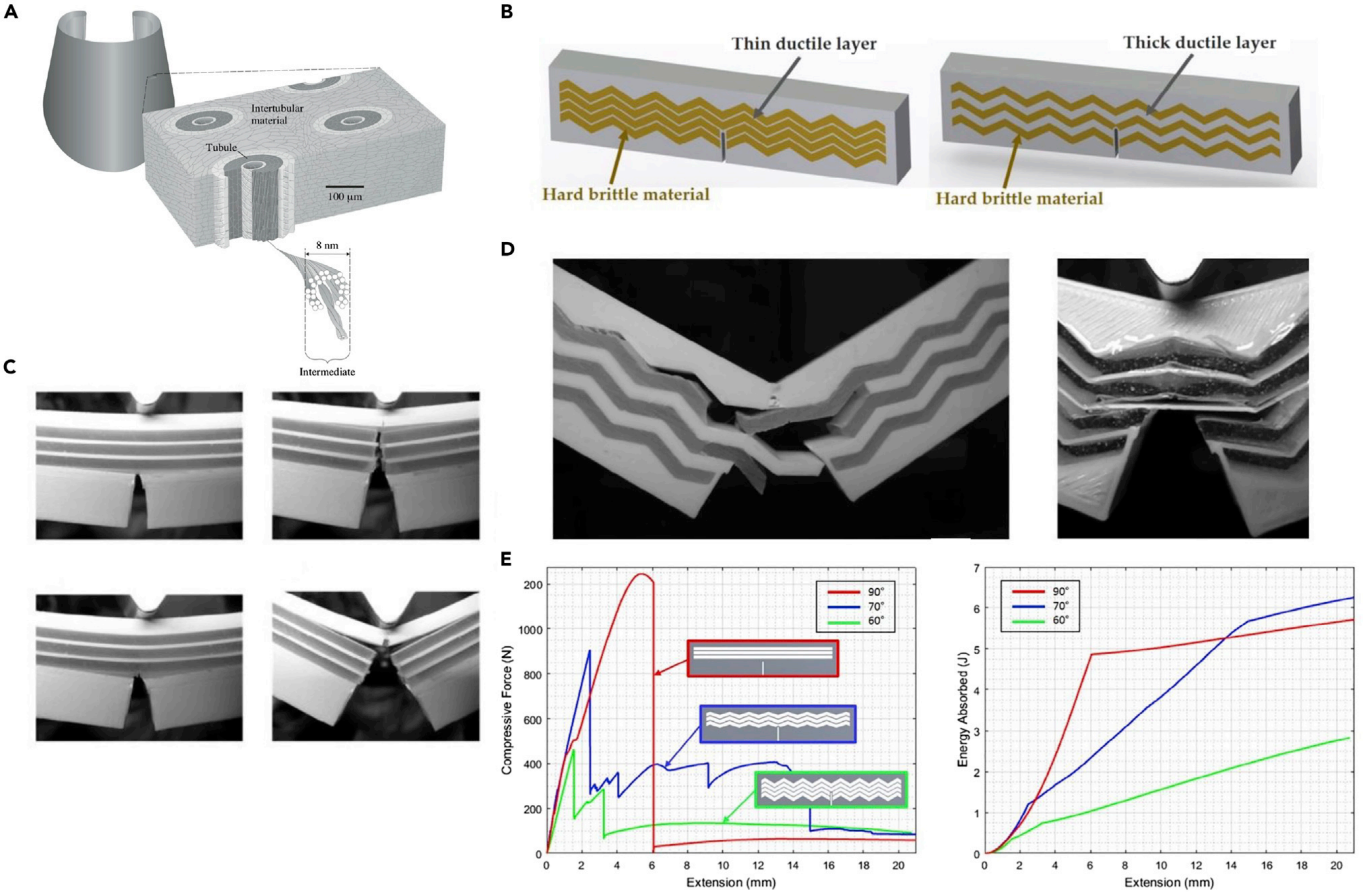
Horse hooves have been a great source of inspiration for tough material designs with fracture control properties (A) A schematic of the horse hoof's micro- and meso-structure showing reinforced tubules embedded in layers of pancake-shaped cells. These cells are filled with IFs. Reproduced with permission (Kasapi and Gosline, 1999). Copyright 1999, Company of Biologists. (B) Schematic showing different epoxy arrangements infiltrated PLA samples inspired by the hoof's layered structure. (C) Crack propagation through flat layered samples before peak stress (top left), at peak stress (bottom left), during failure (top right), and after failure (bottom left). (D) Failure pattern of zigzag layered samples. (E) Schematic showing how cracks interact with a jagged layered structure. (F) Force-extension curve (left) and energy absorption-extension curve (right) of samples with layered structures of different angles. Reproduced with permission (Rice and Tan, 2019). Copyright 2019, Elsevier Ltd.

One of the most common keratinous systems that has been studied for bioinspiration is the hoof wall of horses and bovines (Bertram and Gosline, 1987; Douglas et al., 1996; Huang et al., 2019a; Kasapi and Gosline, 1996, 1997, 1999; Li et al., 2010). Horse hooves hit the ground at a speed of ∼8m/s (Parsons et al., 2011) and can experience impact forces of ∼16.1 N/kg (deceleration of ∼56 g) (Lanovaz et al., 1998; Setterbo et al., 2009). The hoof wall is composed of dead keratinocyte cells that cannot repair themselves yet can survive many regular impacts. This characteristic has made the hoof wall a prime candidate for designing bioinspired materials with high impact resistance and energy absorption capabilities. The hoof wall has an intricate hierarchical structure, depicted in Figure 9A, that has been shown to augment keratin's bulk properties. At the mesoscale, the hoof has hollow cavities (∼40 micrometers in diameter) surrounded by relatively stiff elliptical regions (with a major axis of ∼200 micrometers and a minor axis of ∼100 micrometers) that run parallel to the surface of the hoof wall (Huang et al., 2019a). These tubules are embedded in a lamellar matrix composed of stacked, microscale, pancake-shaped cells (keratinocytes). These two geometries work in concert to provide the hoof with high fracture control (Bertram and Gosline, 1986; Kasapi and Gosline, 1997, 1999) and impact toughness (Huang et al., 2019a; Kasapi and Gosline, 1996).

Rice and Tan (Rice and Tan, 2019) drew inspiration from the lamellar structure found in the horse hoof's intertubular matrix to design improved composite materials. In hooves, the lamellar structure has shown strong retardation of fracture propagation by causing cracks to divert along the interlayer interfaces away from the living tissue at the hoof's interior (Bertram and Gosline, 1986; Kasapi and Gosline, 1997, 1999). To harness this fracture control mechanism for engineered composites, Rice and Tan (Rice and Tan, 2019) manufactured a layered material with alternating soft (ductile) and stiff (brittle) regions, composed of 3D printed polylactic acid (PLA) layers infiltrated with epoxy or resin, as shown in Figure 9B. Their goal was to demonstrate that this bioinspired structure could successfully be utilized in synthetic composites and explore the effects of layer thickness, layer angle, and notch location on crack propagation. Single-edge notched bending tests on monolithic samples of resin, epoxy, and PLA showed that cracks traveled directly through the material with negligible deflection. Similar results were found for samples that contained flat lamellae and thin PLA layers, as shown in Figure 9C. The shear stress near the crack tip initiates debonding between the soft and hard layers; this gives rise to a crack-deflection mechanism similar to those found in hooves. Maximum shear stress develops at 45° to the original notch tip, while the lowest shear stress occurs at 90° to the notch. So, flat layers (layers oriented at a 90° angle to the notch like those in Figure 9C) experience the least debonding and exhibit minimal crack deflection. However, these samples have the benefit of being very stiff and require high peak forces to failure. Figure 9D shows how the introduction of angles into the lamellar structure can affect the crack path through the material. As the angle of the layers relative to the crack tip nears 45°, more shear stress builds up between the soft and hard layers causing the crack to deflect along the interface of the two materials. Figure 9E compares the force-extension curves of samples with layer angles of 60°, 70°, and 90°. Layers at 60° begin to debond at very low forces, while layers at 90° do not exhibit any debonding. Lamellar structures oriented at 70° are an ideal compromise, providing some stiffness and resistance to fracture before absorbing energy by debonding along the zigzag interface. Figure 9E also shows each model's energy absorption curves and indicates that after 14 mm of extension, the 70° model absorbs more energy than the traditional 90° model. One final factor that was found to be very important for this configuration is the layer thickness. When the ductile PLA layer was too thin, the crack fractured through it, and minimal deflection was observed. Higher peak forces and energy absorption were found for thicker ductile layers.

**Figure 10:**
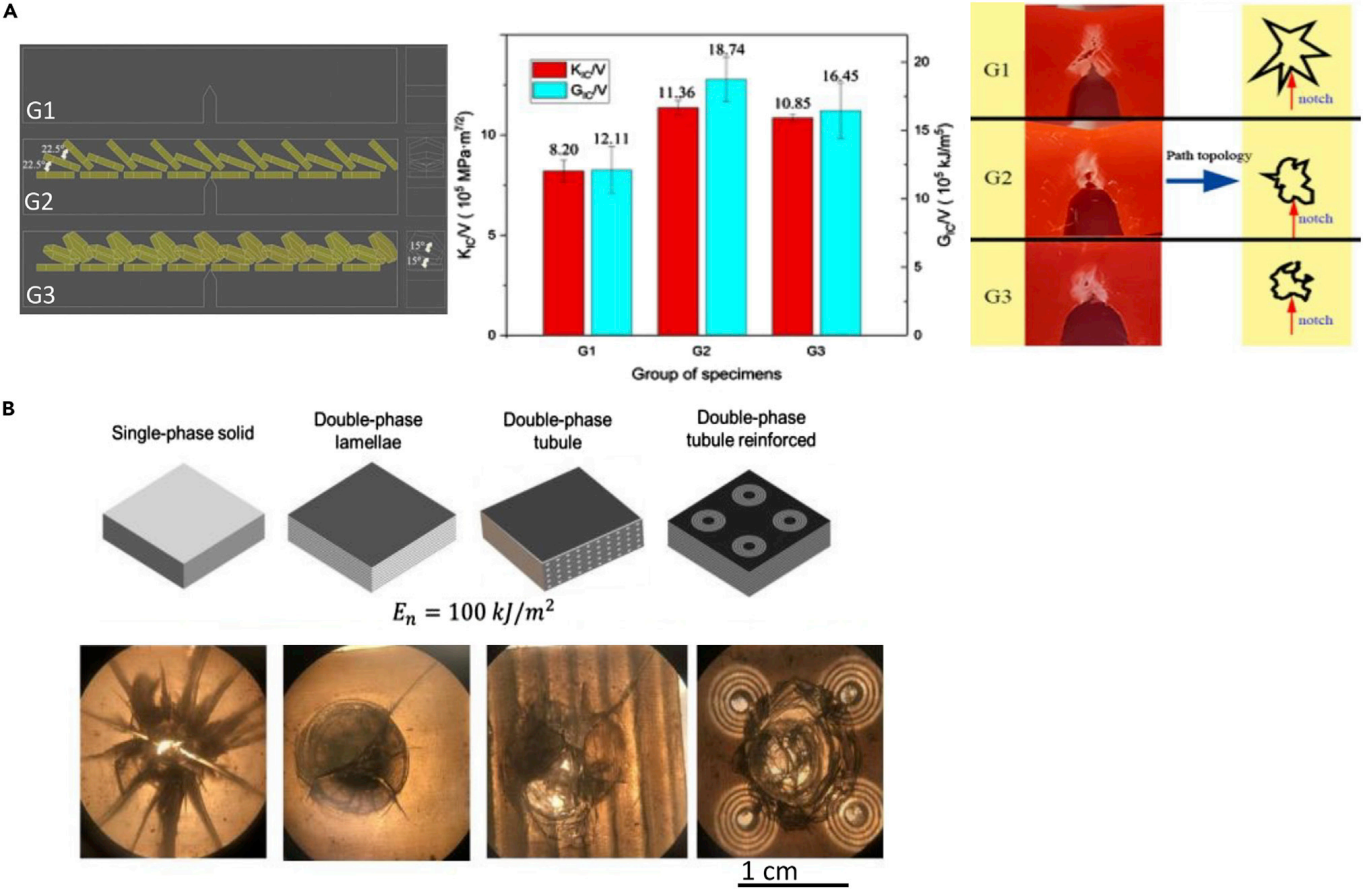
Tubular structures in hooves have attracted significant attention for bioinspired designs (A) Schematic of different tubular arrangements modeled after the hoof with tubules (yellow) represented as hexagonal prisms (left). Graph of normalized K_IC_ for each model (middle), and representative images of the damage zone for each model after testing (right). Reproduced with permission (Wang et al., 2020a). Copyright 2020, Elsevier B.V. (B) Schematic of different models with increasing complexity culminating in double-phase tubules embedded in a layered structure (top). Images (middle) and optical micrographs (bottom) of the different samples after drop tower tests where the impact energy was 100KJ/m^2^. Open Access (Huang, 2018).

Several researchers have also explored the characteristic tubular structures found in hooves. Wang et al. (2020a) 3D printed simplified tubular arrangements based on bovine hooves (Wang et al., 2020a). The tubules were modeled as hollow hexagonal prisms with varying angles that are inspired by the different angles of the intertubular layers found in the hoof. Three different configurations, shown in Figure 10A, were prepared for single-edge notched bending tests. The first model (G1) had no internal structure and was composed of bulk PLA. The second model (G2) had three rows of tubules, each offset from the previous row by 22.5°. The final model (G3) had the same structure as G2, but the tubules had a deflection of 15°. The introduction of tubules significantly improved the mechanical performance of the material with an increase of 39% in K_IC_ and 55% in G_IC_ from the G1 model to the G2 configuration. Figure 10A shows the K_IC_ and G_IC_ results normalized by volume, which indicates the superiority of the G2 design. The samples with tubular elements had a confined fracture pattern, which was given credit for the enhanced toughness and energy absorption of the G2 and G3 models.

Huang et al. (2018) combined reinforced tubular and lamellar structures to understand the impact-resistant synergy that these arrangements provide. Four different models were created using a multi-material 3D printer. These can be seen in Figure 10B. Single-phase samples were made out of stiff and brittle VeroClear. A softer, more ductile polymer called TangoBlackPlus was used to print the black, interlayer regions on the models. Both of these phases are proprietary materials produced by Stratasys, Ltd. Each sample was impacted with 100 kJ/m^**2**^ of energy, and the results are shown in Figure 10B. The single-phase samples failed and fractured into many pieces, while the other three samples all remained intact; only the double-phase tubule reinforced sample prevented cracking from reaching the sample's corners. Optical microscopy images of the damaged samples are shown at the bottom of Figure 10B, where the tubules' crack arresting capabilities can be observed (Huang, 2018).

**Figure 11:**
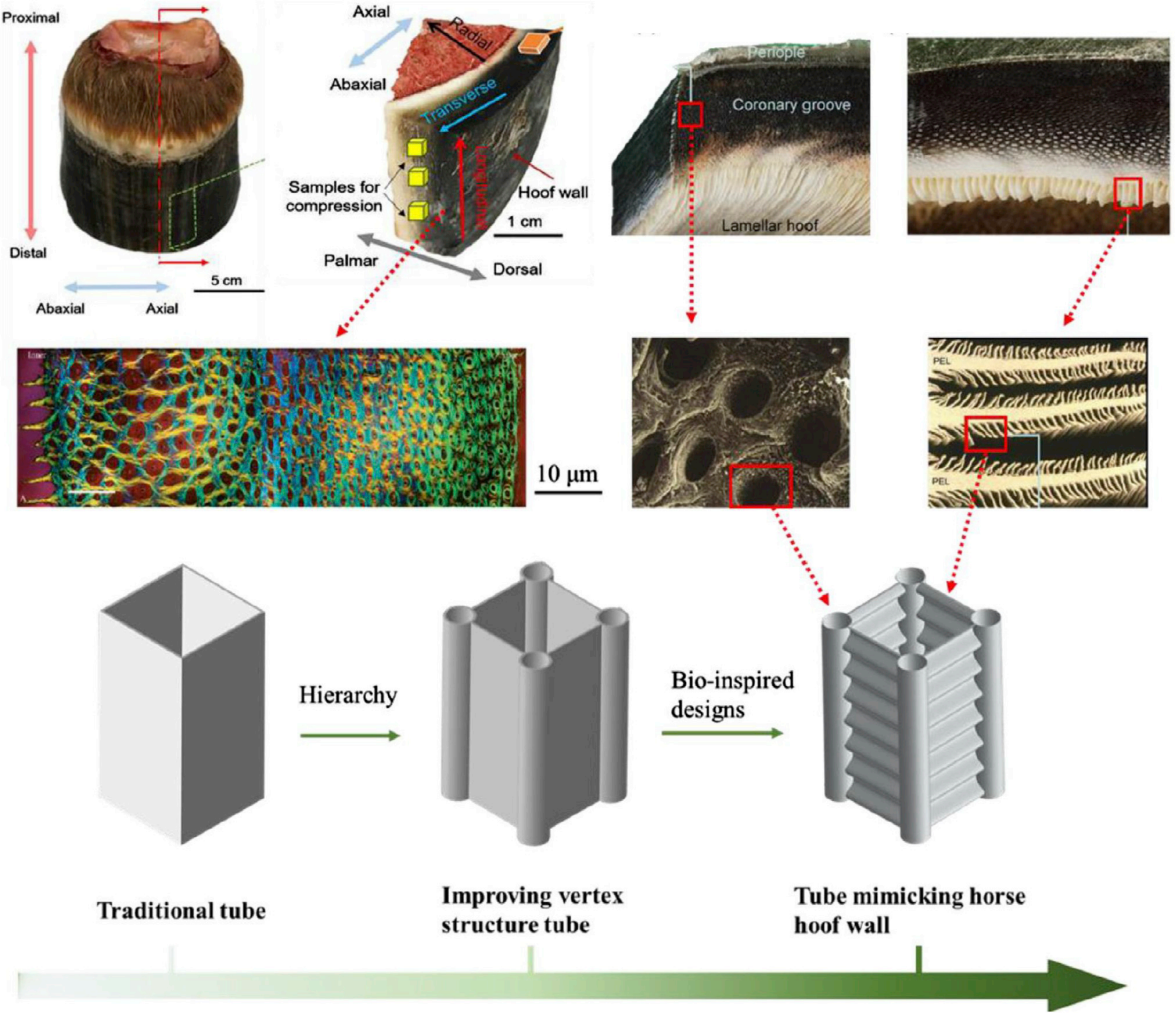
Designs containing structures inspired by the hoof wall have been fabricated to create materials with improved crashworthiness The top two rows of images show the naturally occurring horse hoof, while the bottom row shows designs of increasing complexity that incorporate the tubular and lamellar microstructure of the keratinous hoof sheath. Reproduced with permission (Ma et al., 2020). Copyright 2020, Elsevier Ltd.

Ma et al. (2020) formed tubular structures inspired by the equine hoof wall's architecture to achieve outstanding crashworthiness. As shown in Figure 11, they modified traditional square tubes by replacing the vertices with the unit geometrical structure. The conception of these structures was inspired by the tubular geometry present in the keratinous equine hoof wall. They also modified the side walls to corrugated plates, inspired by secondary epidermal lamella in an inner lamellar layer. The samples were manufactured using the aluminum alloy AA6061-0. Ma et al. (2020) demonstrated that the hoof-inspired geometry (hoof-wall inspired corrugated tube [HCT]) could significantly improve crashworthiness using compression tests and finite elemental analysis. The HCT provided a 269% increase in energy absorption and 124% increase in specific energy absorption over traditional square tubules in compression testing (Ma et al., 2020).

**Figure 12:**
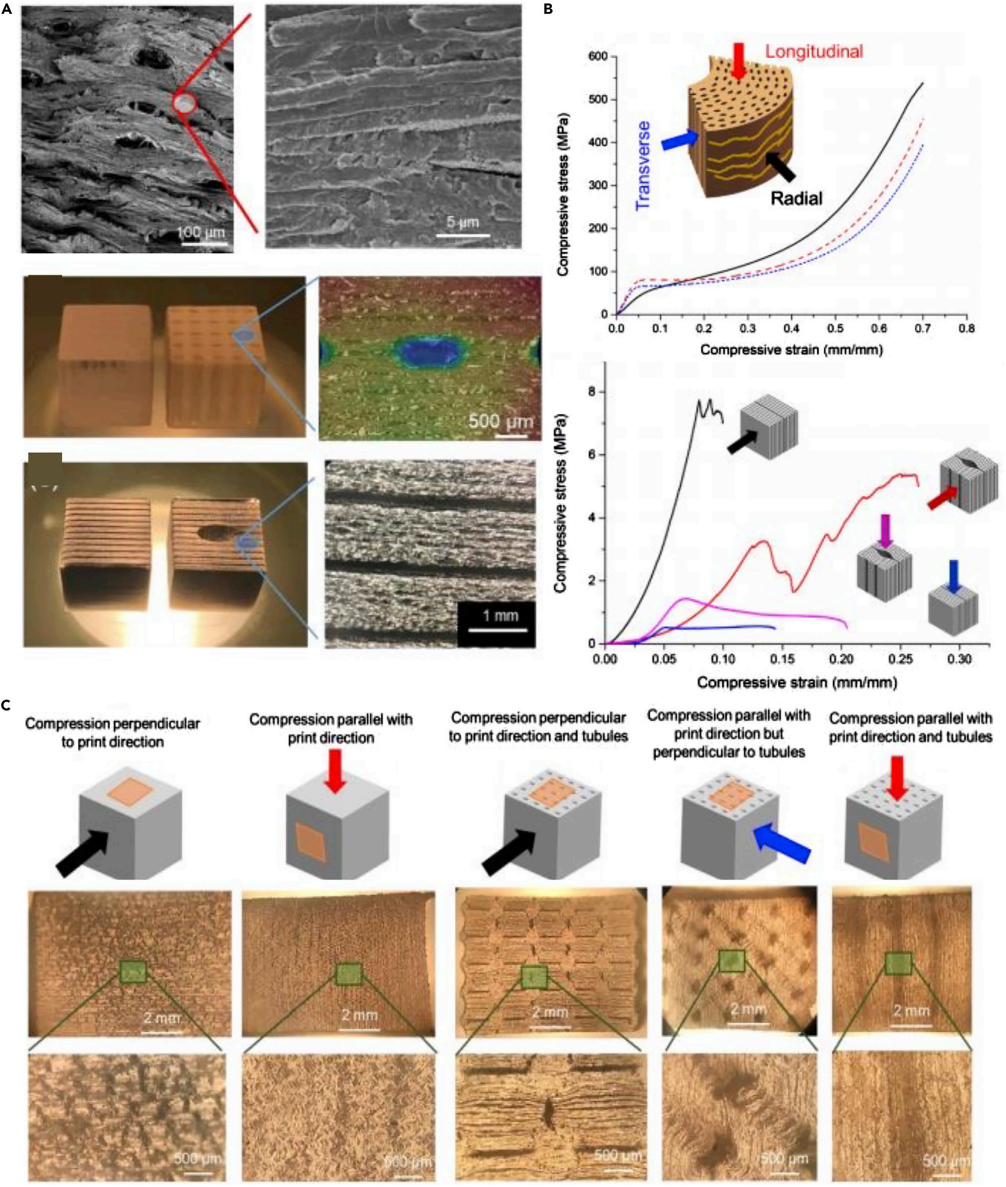
Bighorn sheep horns can endure tremendous impacts and have been the muse for several impact-resistant bioinspired designs (A) The horn's structure (top) with SEM images of its tubular and layered structure. Schematics and images of bioinspired designs with unreinforced tubules embedded in a layered configuration. The layers relative to the tubules' orientation are the opposite of the hooves while the orientation of the tubules to the impact direction is also reversed. (B) Stress-strain curves of the horn and bioinspired samples in different orientations. (C) Images of failure mechanisms of bioinspired samples when compressed in different orientations with respect to print direction. Open Access (Huang, 2018).

Horns have a very similar structure to hooves with hollow tubular elements embedded in lamellar stacks of flat cells. However, the tubules in horns are perpendicular to loading at the impact zone and lack the reinforced region surrounding the tubules that is found in hooves. Figure 12A shows SEM images of the tubular and lamellar structure of the bighorn sheep horn alongside 3D printed models of the horn, including a single-phase block of stiff VeroClear with and without an array of tubules and two-phase lamellar structures (the second phase being ductile TangoBlackPlus). Figure 12B shows how the bioinspired models are compared to horn samples under compression. When samples were compressed with the loading axis parallel to the lamellae, they showed much lower strength. This behavior is due to delamination between the soft and hard phases, similar to the response found in horns. When samples were compressed perpendicular to the tubules, the hollow cavities collapse, leading to a slight decrease in stiffness and strength but an increase in plastic deformation and final compressive strain. Again, this performance mirrored that of real horns, suggesting that this structure could also have good energy absorption capacity under impact (Huang, 2018).

However, 3D printed polymer models of keratinous structures have been limited by their inability to capture these systems' full complexity and mechanical functionality. While the shape of the stress-strain curves of the printed samples and horn samples are similar, Figure 12C shows that their failure mechanisms are quite different. For example, the 3D printed samples developed stress concentrations around the tubules leading to cracking when compressed perpendicular to the tubules. This behavior was not observed in the horn, which was able to distribute stress more uniformly (Huang et al., 2017). Also, when horn samples were compressed parallel to the tubules, tubule buckling was observed. In the 3D printed samples, it was the lamellae that buckled rather than the tubules. These differences are likely due to disparities in material properties between printed and natural samples, the lack of lower-order hierarchical structure in the printed models, and processing restrictions that create weak interfaces and residual stress in 3D printed components. The print direction additionally influences the mechanical response. While 3D printing biomimetic structures have huge potential, this example underscores some of this technique's limitations (Huang et al., 2017).

Huang et al. (2018) also tested the recoverability of compressed 3D printed samples inspired by bighorn sheep horns. Dynamic and quasi-static recovery tests on horn samples showed that, when exposed to water, keratinous materials can regain much of their initial shape after compression. In keratin, this process is highly dependent on hydration, which disrupts the hydrogen bonds within and between the macromolecular chains and allows them to be reformed in a recovered position once the load is released. A similar process can be achieved in synthetic polymers by raising the specimen's temperature over the glass transition temperature. After being compressed to 50% strain, the 3D printed samples were exposed to 62°C for 15 min. Similar to the horn results, damage from compression in the longitudinal and transverse directions was irrecoverable due to lamellae buckling and shear band formation. However, in the radial direction, much of the structure and the stress-strain curve was recovered in subsequent compression cycles, suggesting that keratinous materials can also provide a structural blueprint for shape recovery materials (Huang, 2018).

**Figure 13:**
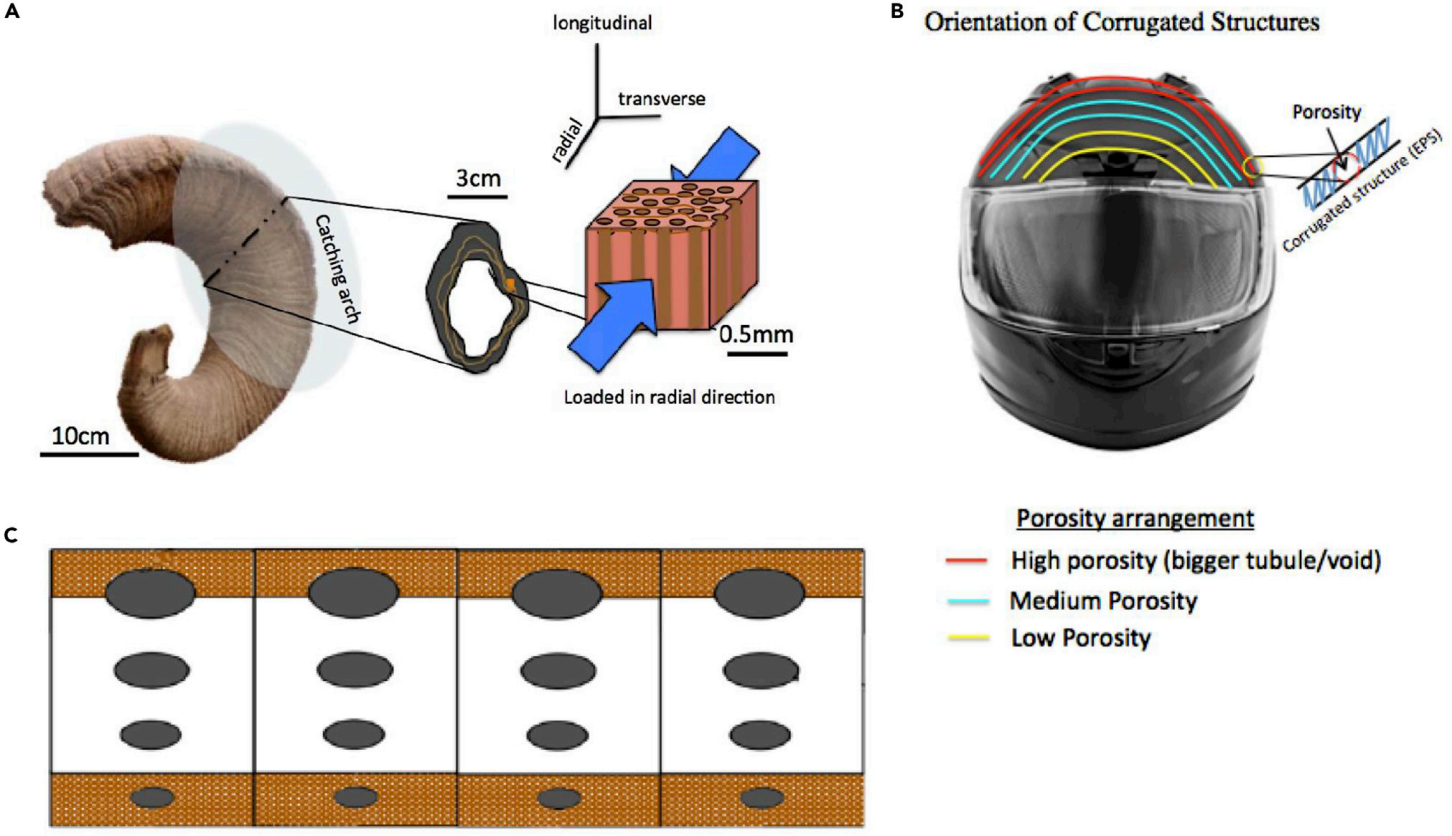
Bighorn sheep horns absorb tremendous impacts in nature, so researchers envision helmets inspired by the horn's microstructure (A) Visualization of the hierarchical structure with an emphasis on the microstructure of the bighorn sheep horn. (B) Conception of a helmet with a gradient in tubular porosity between the interior and exterior. (C) Cross section of the protective tubular region showing a variation in tubule size through the helmet's thickness. Reprinted with permission (Kassar et al., 2016).

Kassar et al. (2016) produced foam liner material for motorcycle helmets inspired by the microstructure of horns. Helmets and horns both have an outer structure that is mainly responsible for energy absorption during impact. Soft inner tissue that distributes the load increases the deceleration distance and thus protects the head. Following a similar principle, they designed foams with varying tubular porosity. As observed in horn structures, the tubules' porosity was varied from 0%, near the head, to 10% in the middle and ∼30% on the outer shell (Kassar et al., 2016). This spatial change in porosity is a classic example of a gradient structure, one of the hallmarks of biological materials (Liu et al., 2017). Figure 13 shows the bioinspired design. To assess the design, modified drop tower tests according to “United Nations Economic Commission for Europe Standard” for motorcycle helmets ECE 22.05 were performed using the foam manufactured by EPS material. The design was able to meet safety thresholds far below the limits stipulated by the ECE 22.05 motorbike helmet testing standard.

The above efforts have taken bioinspiration from microscale structural elements of hooves and horns. However, Sun et al. (2014) designed rear under-run protection devices (RUPDs) for heavy trucks inspired by the macroscale geometry of the sheep horn. The RUPD prevents the entry of small-scale vehicles under the rear end of the heavy truck. The design was analyzed using a finite element analysis. The authors concluded that, compared to the normal RUPD of the same thickness, the bioinspired design could provide better protection when rear-end accidents happen; this is due to its enhanced energy absorption and structural strength (Sun et al., 2014). Zhang et al. (2008) took inspiration from buffalo hooves to design impellers for a paddy field. They studied the buffalo hooves' curvature that allows them to maneuver through the field with relative ease. The impeller designed with similar curvature has a 38% increase in pull force and was more efficient than standard blades (Zhang et al., 2008).

**Figure 14:**
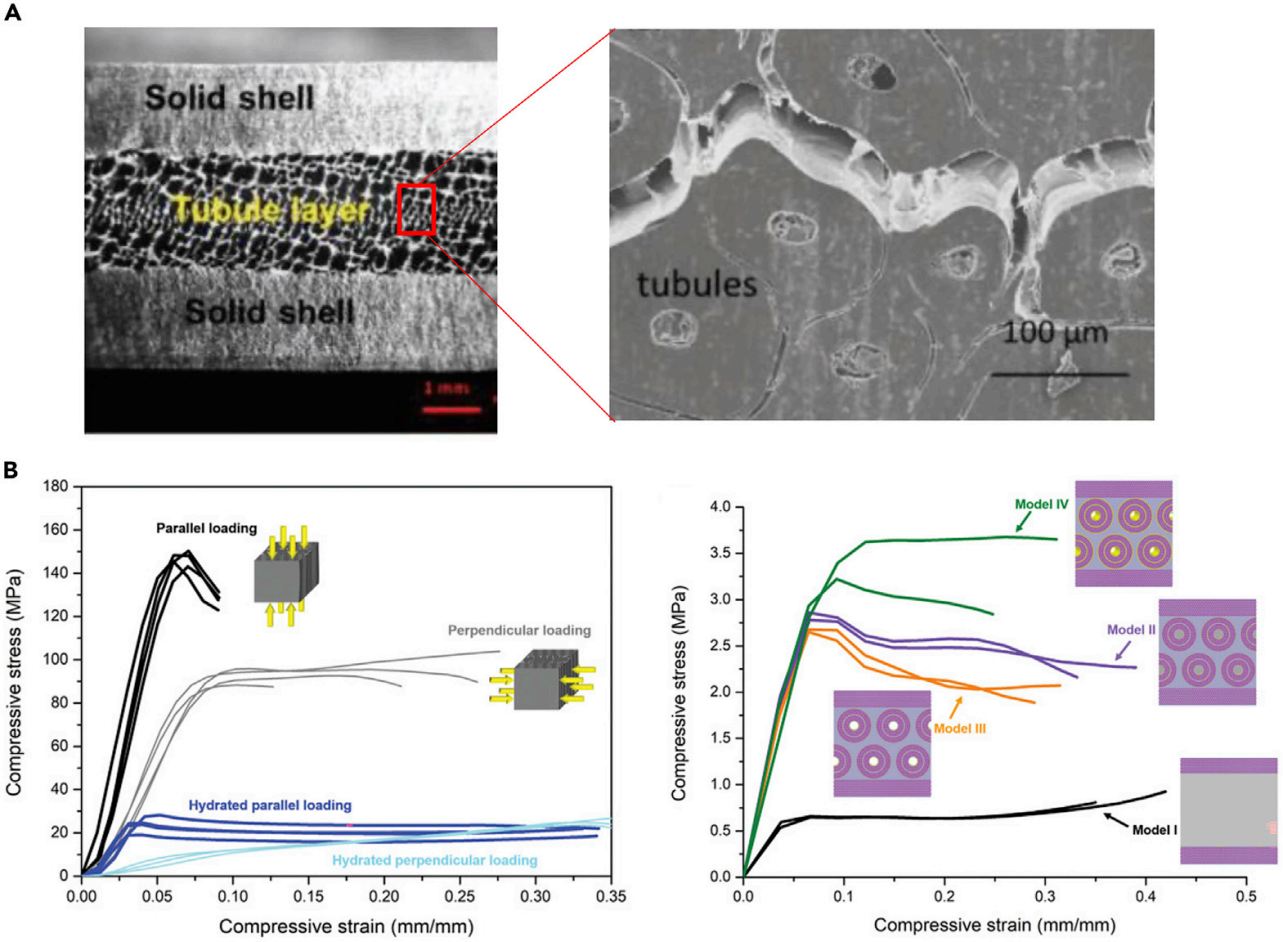
Whale baleen is a part of the filter-feeding apparatus of baleen whales and is able to withstand high stresses and impacts from fish that get sucked into the whale's mouth. Bioinspired models have shown that the structure of the baleen helps endow it with admirable properties (A) Image of a cross section of whale baleen showing the tubule layer sandwiched between a solid shell of keratin. (B) Stress-strain curves of the baleen in each orientation showing significant differences in response based on loading direction. Stress-strain curves of the bioinspired models, indicating the design's superiority with all of the features incorporated in tandem in model iv. Reproduced with permission (Wang et al., 2019). Copyright 2018, WILEY-VCH Verlag GmbH & Co. KGaA.

Baleen is the filter-feeding system found in the oral cavity of baleen whales, some of the largest animals on the planet, and is composed of highly mineralized keratin. To withstand the forces associated with filter-feeding, some whales have evolved baleen with complex structures that provide remarkable fracture toughness. The baleen plates contain a tubular sandwich structure that can be seen in Figure 14A. The tubular region has a structure that is reminiscent of hooves but has a much higher mineral content that arises from hydroxyapatite nanocrystals embedded among the keratin IFs. The sandwich structure, composed of a solid shell around the tubular zone, provides high flexural stiffness and strength relative to the material's weight. Much like the tubule lamellae found in hooves, the concentric layered arrangement around the hollow cavities serves to deflect cracks and increase fracture toughness. This structure is highly anisotropic. The differences that arise from different loading directions can be seen in Figure 14B. Loading parallel to the tubules gives higher Elastic modulus but less ductility than the loading perpendicular to the tubules. This anisotropy has a profound effect on fracture toughness (Wang et al., 2019).

Four 3D printed models were fabricated to investigate the role that each of these features plays in the baleen. The most complex model (model IV) printed using three different materials most closely represents baleen. The mineralized lamellae were simulated using a stiff polymer and the matrix using a ductile polymer, while a polymer of intermediate stiffness represented the unmineralized lamellae filaments. Each successive model adds a new design element. Model I contains just a sandwich structure of soft material between two stiff layers; model II adds the concentric filament structure; model III includes the hollow cavity at the center of tubules. Model IV combines all of these features with the stiff lamellar rings shown in yellow. The addition of filaments raised the samples' stiffness, while the hollow cavities slightly decreased the sample strength at strain rates of 0.28 s^−1^ and 10^−4^ s^−1^ but increased it at strain rates of 10^−2^ s^−1^. The addition of the stiff lamellar rings unsurprisingly increased the models' stiffness and strength and led to significantly more strain-rate stiffening and strengthening. These phenomena were also observed in the natural baleen. Wang et al. (2019) concluded that model IV provides the best mechanical performance showing that the features found in keratinous whale baleen can be utilized as beneficial structural design elements (Wang et al., 2019).

In summary, bioinspired research on mechanical keratinous tissue has focused on several features: tubules (as found in the hoof, horn, and baleen), lamellar structures (found in all keratinous materials), and macroscale geometry (like hoof curvature or horn shape). When composite materials incorporate tubules or lamellae, they find improved fracture toughness due to crack interactions at these structures' interfaces. Similarly, macroscale geometries are practical but largely unexplored avenues of inspiration for specific functions like impellers or bumpers.

### Thermal insulation

Keratinous systems are some of nature's best insulation by virtue of their elaborate structures that trap air. Many synthetic fibers are more inherently resistant to heat transfer. However, with their hierarchy of air-trapping features, natural keratinous systems are still some of the most superb thermal insulators. The popular and unsurpassed down jackets use feathers. As a result, researchers have tried to recreate these natural insulators' configurations in engineered materials to harness their desirable thermal capabilities.

**Figure 15:**
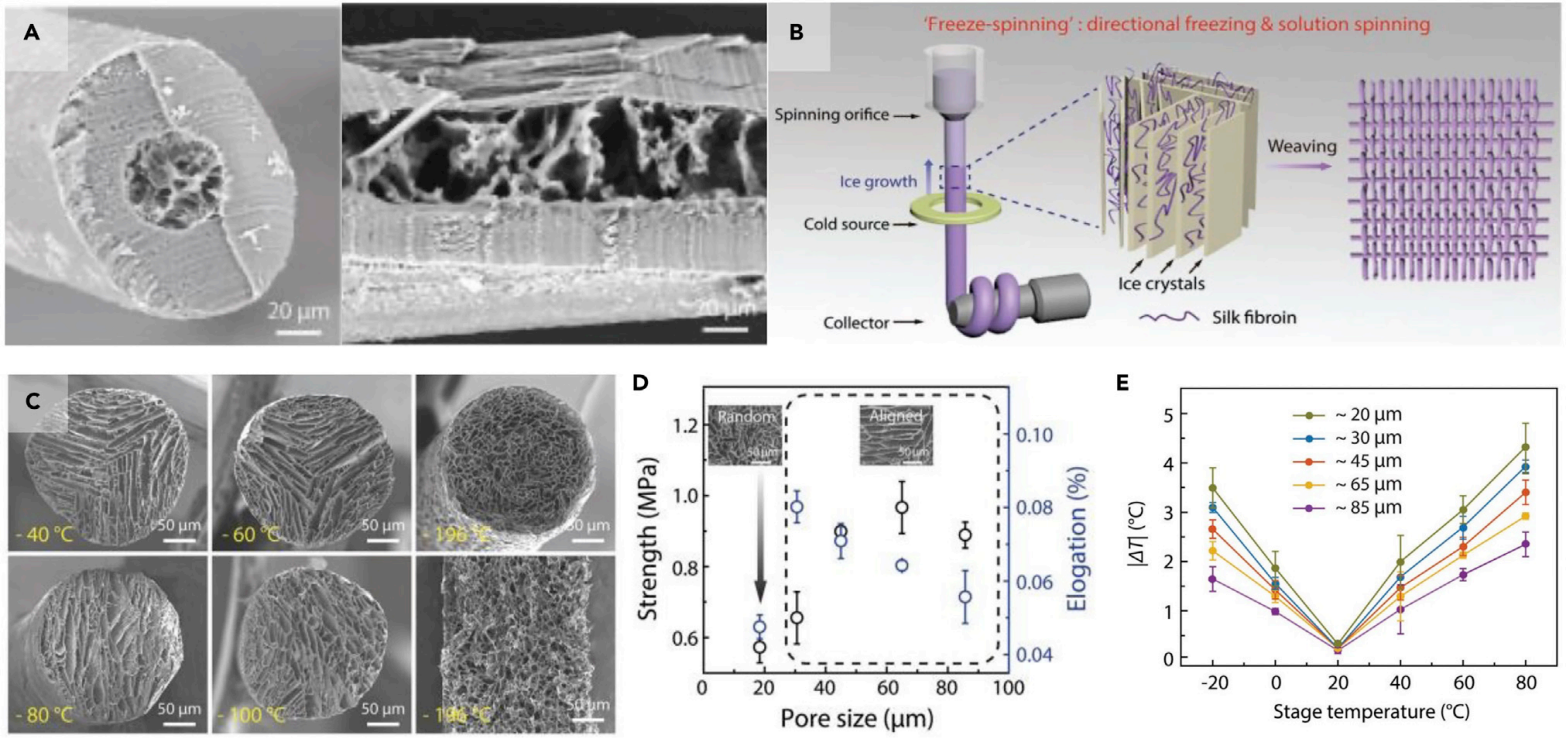
Polar bears can survive in some of the harshest environments on earth, largely due to their warm fur. Bioinspired models based on porous hairs have been fabricated to harness the remarkable thermal properties exhibited by polar bear hair (A) SEM images of polar bear hair radial (left) and longitudinal (right) cross sections. (B) Design set up for freeze spinning system used to fabricate bioinspired polar bear hairs fibers. (C) SEM images of bioinspired hair cross sections fabricated at different temperatures. (D) Plot of average pore size vs. fiber strength in the bioinspired fibers. (E) Plot of difference in heat between the top of fibers and bottom of fibers with varying average pore size when placed on a heated stage over a range of temperatures (−20°C - 80°C). Reproduced with permission (Cui et al., 2018). Copyright 2018, WILEY-VCH Verlag GmbH & Co. KGaA.

Some organisms, like polar bears and penguins, can thrive in the most extreme conditions on earth due to their keratinous thermal protection (Jia et al., 2017; Metwally et al., 2019). Polar bear hairs consist of a hollow porous interior that provides superior thermal properties surrounded by a shell of aligned fibers, which supplies mechanical stability. SEM images of these hairs are shown in Figure 15A. Individual hairs are approximately 200 micrometers in diameter, while the interior pores measure 15-20 micrometers across. The length scale of these pores is significant because it allows the hairs to trap substantial amounts of air, providing a thermal buffer between the bear's living tissue and the surrounding arctic temperatures that can reach as low as −45°C.

Since 3D printing cannot manufacture architectures on the scale of micrometers, Cui et al. (2018) used freeze spinning to create bioinspired synthetic fibers that could mimic the polar bear hair. This process is similar to freeze-casting in that it harnesses directional ice crystal growth to create a porous lamellar structure within an aqueous solution. However, freeze spinning performs this technique within a stable, extruded liquid wire. Once the wire is frozen, the material is freeze-dried to preserve the intricate microstructure formed by the ice crystals, and the completed porous fiber can be woven into a textile (Cui et al., 2018). This process is visualized in Figure 15B.

As with freeze-casting, several parameters can be adjusted to control the production, such as solution viscosity, extrusion speed, and freezing temperature. For the latter, Cui et al. (2018) found that the temperature at which the ice crystals are formed can be used to control the pore size and orientation in the fiber, as shown in Figure 15C. As the temperature is lowered from −40 C^o^ to −196 C^o^, more ice crystals are formed, but the freezing process occurs quickly, giving the crystals less time to propagate through the solution. The result is more numerous pores that are smaller in size. When the fiber is frozen in liquid nitrogen, a random porous network is produced, but when crystals are formed at higher temperatures, the pores align in the crystal growth direction. The pores' alignment and size have a significant effect on the fibers' tensile properties, as seen in Figure 15D. Aligned pores provide better strength and elongation than a random porous network (Cui et al., 2018).

In comparison, fibers with larger pores tended to have higher strength but lower average elongation than fibers with smaller pores. Smaller pores, however, provide better thermal properties. This behavior was determined by heating fibers with different pore sizes on a stage and measuring the temperature on fibers' surfaces using IR images. These results are summarized in Figure 15E. This biomimetic material also showed promising results for thermal cloaking and, when embedded with carbon nanotubes, electro heating (Cui et al., 2018).

Feathers are among the most ubiquitous materials used as thermal insulators due to their extreme lightweight and durability. Different types of feathers are distinguished by their structure and location on the bird: contour (body feathers) and plume (down feathers). Down feathers are primarily responsible for thermal insulation, which is attributed to their hierarchical foam-based structure creating large surface areas for trapping heat. Some academics have posited that Eiderdown, in particular, is the most thermally insulating natural material in the world (Kasturiya et al., 1999). Down benefits from an impressive strength-to-weight ratio (Gao et al., 2007; Havenith, 2010), compressibility (Gao et al., 2010), and compression recovery (Martin, 1987), making it invaluable as bodily insulation in extreme environments. The first use of down jackets was seen in expeditions to Mount Everest in 1922 and by 1933 in down sleeping bags, which have become a staple of mountaineering in the harshest of climates. While this application of keratinous tissue is hardly bioinspiration, this review would be incomplete if it did not mention the pervasiveness of feathers in a vast range of textiles from common bedding to elite sub-zero clothing (Fuller, 2015). Even before down became popular, other keratin sources such as wool and animal fur have played a dominant role in the human race's ability to inhabit some of the coldest regions on earth.

One of the driving enterprises of the industrial revolution was the production of textiles. With such vast commercial implications, research on manufacturing cheap, synthetic fabrics with properties similar to wool and fur has been evolving for centuries. Modern clothing is often a mix of natural materials such as wool or cotton and synthetic fibers like polyester. In some cases, natural fibers have been replaced entirely. Examples include synthetic cashmere, which is usually a combination of rayon, nylon, and polyester, and fleece, typically composed of PET. Ultimately, many of our modern textiles are bioinspired materials that are attempting to replicate the success of traditional but expensive, labor-intensive keratinous systems.

In summary, keratinous materials' thermal insulation revolves around hierarchical surface texture or internal pores that are meant to trap air pockets and create a buffer between the animal and its surroundings. Efforts to recreate these structures using synthetic materials have been quite successful, highlighting that this is a fruitful area of study.

### Reversible adhesion

Reversible or non-destructive adhesion allows for repeated attachment and detachment cycles that do not damage the substrate. Nature employs a variety of reversible adhesive strategies: mechanical interlocking, friction, chemical bonding, dry adhesion (i.e., van der Waals), wet adhesion (i.e., capillary), and suction (i.e., pressure differential). Often, organisms will use a combination of the above attachment methods to adhere to surfaces successfully. These processes are strongly dependent on the environment (predominantly wet vs. dry and smooth vs. rough). Mechanical interlocking, friction, dry adhesion, and wet adhesion are strongly dependent on having nanostructured surfaces. The hierarchical nature of keratin lends itself well to forming nanostructured and intricate designs. While the field of reversible adhesion is extensive (Arzt et al., 2003; Gorb, 2008), our focus here is on materials inspired by keratin-based systems to highlight the diverse functionality that keratin offers. We will focus on the mechanical attachment found in the feather vane and dry adhesion found in gecko setae and their respective bioinspired designs. Claws and talons use a more conventional design principle, a relatively large hook, and will not be treated here.

**Figure 16:**
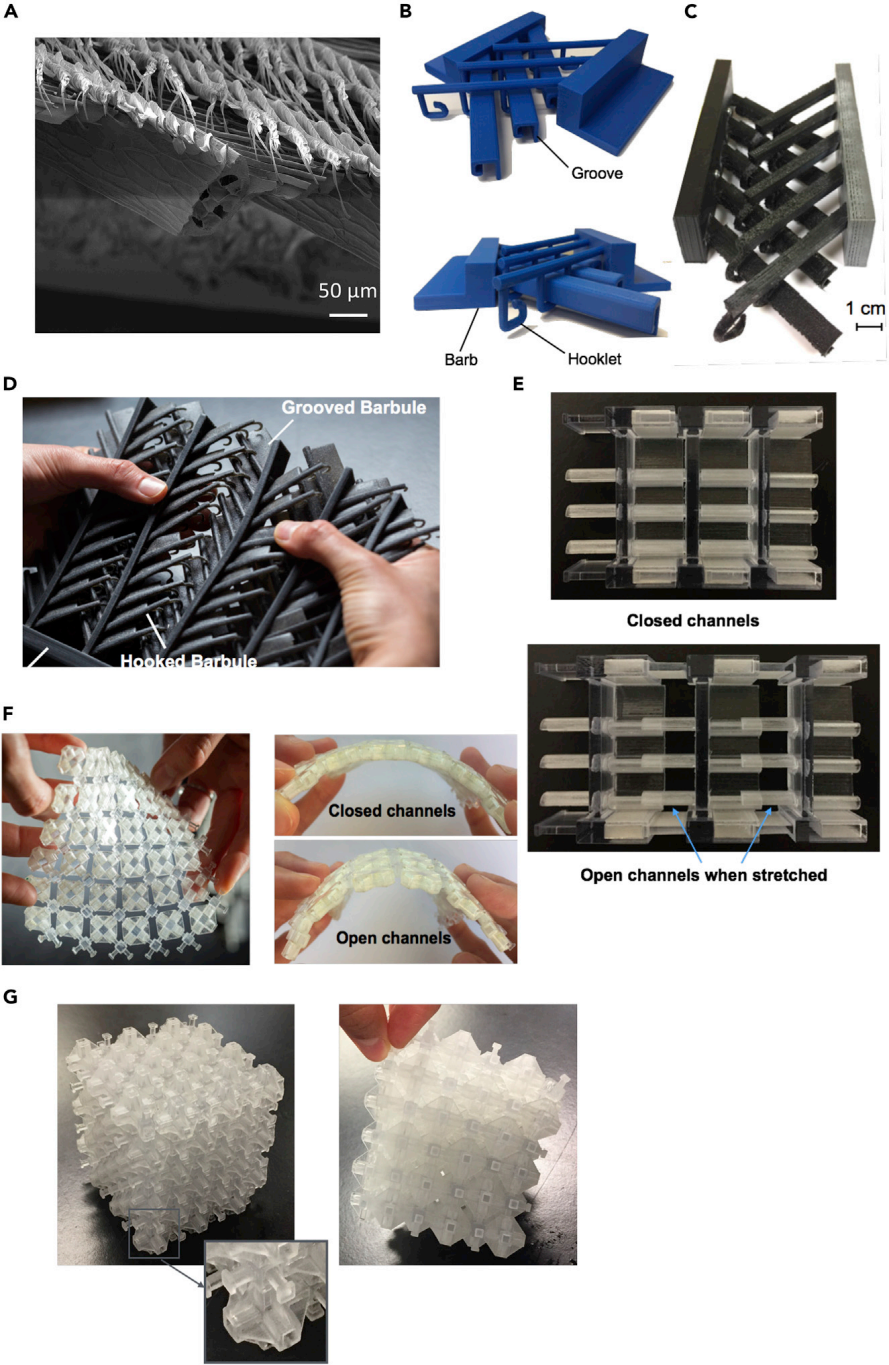
Progression of bioinspired designs based on the attachment mechanism found in the feather vane (A) SEM micrograph of the feather vane showing a branched network of barbs, barbules, and hooklets. (B) First hook and groove-inspired sample. (C) Modified hook and groove structure with a closer match in stiffness to the actual feather vane. (D) Advanced replication of the feather vane to incorporate membrane flaps for directional permeability. (E) The first groove-only unidirectional sliding structure. (F) Two-dimensional sliding structure, which shows textile-like behavior. (G) Cubic sliding structure which provides tailored stiffness in three dimensions. Adapted with permission (Sullivan et al., 2019). Copyright 2019, Elsevier.

The feather vane is directionally permeable, which effectively helps it capture air for lift (Alibardi, 2007; Sullivan et al., 2017a). This mechanism is controlled by the branching barbs' geometry and stiffness and interconnecting barbule network, which ultimately forms the feather vane (Alibardi, 2007; Sullivan et al., 2017a). Barbs, which branch from the rachis, are further branched into barbules. The barbules have hooklets (hamuli) on their extremities, which fit into the neighboring barb's groove, creating a highly ordered lattice of interconnected adjacent barbs (Figure 16A). Having multiple hooklets increases both the adhesion and the probability that two neighboring barbs will stay connected. The interconnected network of the feather vane, provided by this adhesive mechanism between the barbs and barbules, is credited as the essential element that allows birds to achieve flight (Sullivan et al., 2017a).

Several 3D printed bioinspired designs based on the reversible adhesive mechanism of the feather vane have been developed by Sullivan et al. (2019). These 3D printed structures not only serve to understand better the mechanisms operating in the feather but extend beyond the scope of the intended function in nature to suggest innovative solutions for deployable structures, next-generation chain mail, and smart foams. The initial interlocking barbule bioinspired design was intended to mimic the attachment mechanism by scaling the dimensions to an appropriate size for 3D printing and mechanical testing. The first design in Figure 16B helped demonstrate the feather vane's adherence through hooks and grooves that slide along each other. This feature is similar to the mechanism found in Velcro but is more organized and directional. While the first design served as a simplified model, the 3D printed material used was much stiffer than the feather vane. It did not accurately mimic the feather vane's elasticity and ability to re-adhere. The second model attempted to reduce the elastic mismatch by printing with a more flexible material which helped to elucidate how the material properties and the hook's geometry can enable sufficient adhesion that is damage-tolerant and can re-adhere readily (Figure 16C). Development of subsequent designs based on the barb and barbule interaction further increased in complexity to represent the feather vane with the inclusion of flaps that act as one-way valves (Figure 16D). These models suggest that two existing modes allow for tailored air permeability: (1) membrane flaps allow air to flow through space between barbules dorsally but not ventrally, and (2) the sliding of hooks along the grooves offers expansion within the feather vane (when hooks are closer to the base of the groove the vane is tighter, i.e., less permeable than when the hooks are at the tip of the groove). The purpose of this effort was to offer a simplified visualization of the complex nature of reversible adhesion and directional permeability in the feather vane (Sullivan et al., 2019).

The subsequent iterations of designs expanded beyond just mimicking the feather vane by extracting fundamental design principles to optimize the interplay of tailorable, expansive materials. The ideas involved removing the hooks, which were shown to be the weakest point of adhesion from previous designs (Figures 16A–16D), and creating an exclusively grooved-based structure that had stoppers at the end to prevent complete detachment (Figure 16E). In the first groove-based structure, sliding was only able to occur in one direction. In this direction, sliding enabled an increase in flexibility while the perpendicular direction remained rigid. The design was further altered to allow sliding in both directions, which led to textile-like behavior when stretched open (Figure 16F). Finally, a cubic structure was developed, which allowed for sliding and manipulating the modulus in all three dimensions (Figure 16G). This progression in development highlights the importance of bioinspired design as a creative process reaching beyond the limitation imposed on nature to develop innovative materials.

The gecko setae are a most striking example of reversible adhesion in nature. Over the past two decades, these keratinous nanopillars have stirred up a tremendous amount of scientific interest, leading to the publication of hundreds of research papers and enough articles to be the topic of their own review (Boesel et al., 2010; Jeong and Suh, 2009; Li et al., 2019; Niewiarowski et al., 2016; Russell et al., 2019; Stark and Mitchell, 2019; Wang et al., 2020b; Zhou et al., 2013a). However, interest in the gecko stretches back through the past century. Piquantly, geckos' mysterious ability to climb vertical walls and even to hang upside down on ceilings was correctly interpreted in 1902 by Franz Weitlaner (Kroner and Davis, 2015). Since then, great strides in bioinspiration have come with vast amounts of research, and many groups have succeeded in making reversible dry adhesives based on the gecko setae structure. Here, we will only broadly cover this burgeoning area of study.

**Figure 17:**
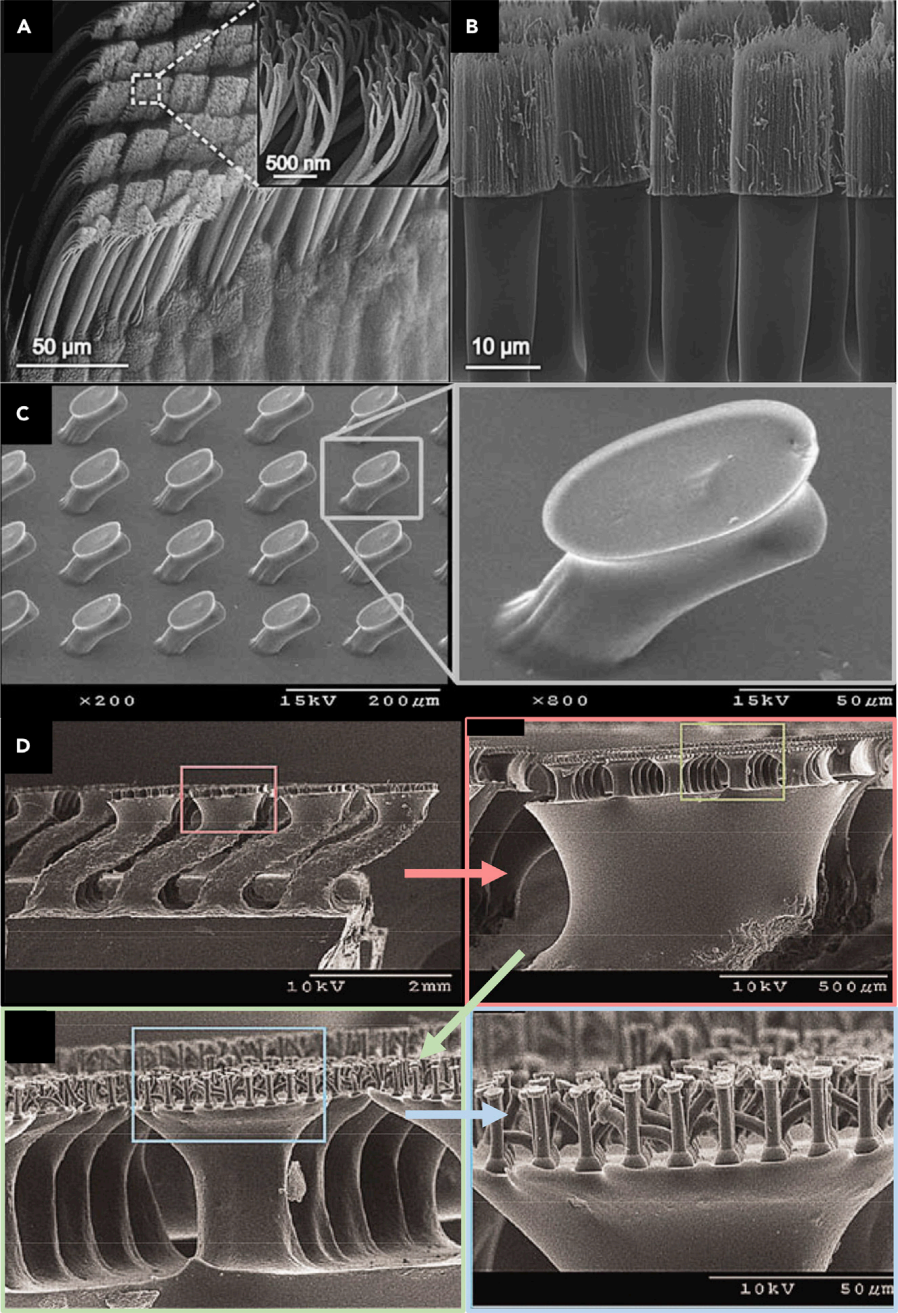
Geckos use van der Waals forces generated by densely packed setal arrays on the feet to climb even the sheerest surfaces. Many researchers have attempted to replicate this structure to create reversible, dry adhesives (A) SEM image of the branched gecko setal array. The inset image shows the split-fiber endings with tilted, spatula-shaped tips (Rong et al., 2014). (B) SEM image of synthetic gecko-inspired adhesive composed of polymer micropillars with densely packed carbon nanotubes glued to the end. Open Access (Rong et al., 2014). Copyright 2013, the authors. (C) SEM image of bioinspired, tilted micropillars composed of polyurethane that mimic the gecko setae's directional gripping strength. Reproduced with permission (Murphy et al., 2009a). Copyright 2009, Wiley-VCH Verlag GmbH & Co. KGaA. (D) SEM images of three hierarchical tiers of mushroom-shaped pillars composed of polyurethane that mimic the hierarchical branched structure found in the gecko pad. Reproduced with permission (Murphy et al., 2009b). Copyright 2009, American Chemical Society.

The gecko's adhesive pads utilize van der Waals forces and, to a lesser extent, capillary forces generated by the hierarchical broom-like geometry of setal arrays (Li et al., 2019; Russell et al., 2019). The setae found on the gecko adhesive pad are arranged on lamellae and branch into hundreds of individual spatula-shaped tips (typically referred to as spatulae), as shown in Figure 17A. van der Waals forces require extremely close contact (<10 nm) to generate a significant force, and this is accomplished by the flexible, branched nanostructure of the gecko pad. As the setae divide into smaller subdivisions with higher aspect ratios, their effective elastic modulus decreases, allowing them to conform easily to smooth and rough surfaces (Russell et al., 2019). In the aggregate, the spatulae generate significant adhesive forces in the normal direction and frictional forces in the lateral direction, both of which are vital to the locomotion of the gecko (Boesel et al., 2010). Further, these fine subdivisions have the added benefit of confining crack propagation if a single seta begins to fail (Li et al., 2019).

There are several additional characteristics of the gecko pad that make them particularly alluring to researchers. One of the gecko pad's most enticing features is its controllable and reversible adhesion, allowing it to be reused and not leaving behind any residue on the locomotive surface. The setal spatula-shaped tips attach to a surface when the gecko pulls its toes downward and inward, creating a small pulling angle between the setae and the surface. To detach from a substrate, the gecko pushes its toes upward and outward; this has the dual effect of increasing the pulling angle past the critical detachment angle (∼30°), while squeezing the setae to increase their effective elastic modulus and decrease their conformability to the surface (Li et al., 2019; Russell et al., 2019; Tian et al., 2006). This process can be actualized in a matter of milliseconds allowing for rapid, reversible adhesion. Other unique properties provided by the branched setal arrangements include self-cleaning characteristics and adhesion in challenging environments like underwater and on surfaces with different polarity and roughness (Russell et al., 2019; Stark and Mitchell, 2019).

Several research groups have hypothesized that the self-clean capabilities of the gecko-pad arise directly from the nanostructure of the setal configurations. Hansen and Autumn (2005) suggest that the primary reason for self-cleaning is the energetic disequilibrium between the substrate and the setae, but state that other factors like locomotion, particle rolling, and particles wedging between the setae could play a role (Hansen and Autumn, 2005). Follow-up studies have confirmed that each of these mechanisms improve self-cleaning (Hu et al., 2012; Mengüç et al., 2014), particularly when particles bond more strongly to the setae than the substrate. Xu et al. (2015) postulated that the dynamic motion of gecko toes (referred to as digital hyperextension) allows geckos to tune the pull-off velocity of the setal arrays (Xu et al., 2015). Since the adhesive force between dirt particles and the substrate is velocity-dependent, and the force between particles and the setae is largely velocity-independent, increasing the pull-off rate can dislodge bonded particles from the surface of the toe pads. This velocity-controlled self-cleaning technique was applied to synthetic biomimetic materials with great success, achieving an ∼80% chance of particle detachment at high velocities (>1000  μm s^−1^) compared to the 0-40% chance of detachment beneath this threshold.

Many different approaches (Arzt et al., 2021) have been utilized to fabricate nanostructures capable of generating dry, reversible adhesion inspired by the gecko setae. These include soft lithography (Crosby et al., 2005; Lamblet et al., 2007; Peressadko and Gorb, 2004), injection molding (Gorb et al., 2007a, 2007b; Gorb and Varenberg, 2007; Varenberg and Gorb, 2007), hot embossing (Hu et al., 2014), photolithography (Aksak et al., 2007; del Campo and Greiner, 2007; Davies et al., 2009; Northen and Turner, 2005, 2006), plasma etching (del Campo and Greiner, 2007; Jeong et al., 2009), electron beam lithography (Geim et al., 2003), carbon nanotubes (an example of which is shown in Figure 17B) (Hu et al., 2013; Qu et al., 2008; Rong et al., 2014; Yurdumakan et al., 2005), nanodrawing (Jeong et al., 2006), micro/nanomolding (Glassmaker et al., 2004; Greiner et al., 2007; Mahdavi et al., 2008; Sitti and Fearing, 2003), dip-transferring (Murphy et al, 2009a, 2009b), two-photon lithography (Hensel et al., 2018), nanoimprint lithography (Raut et al., 2018), and many more. As research groups have aimed to mimic the gecko pad's intricate structure more closely, the complexity of their fabrication processes has increased. Early techniques focused on only manufacturing a dense network of nanopillars. However, these studies showed that other design parameters need to be considered to truly capture the gecko pad's functionality.

For example, the natural setae are tilted, which creates much larger shear forces in the gripping direction than in the non-gripping direction, effectively enhancing the gecko pad's reversible adhesion (Boesel et al., 2010). Figure 17C shows tilted polyurethane fibers fabricated via inclined exposure and dip coating to capture this parameter. Another essential variable for gecko-inspired adhesives is the shape of the tip of the nanofibers. Many different arrangements have been investigated, but mushroom-shaped tips have proven to be the most successful design (Russell et al., 2019).

del Campo et al. (2007) compared biomimetic arrays with various pillar shapes and found the mushroom configuration to have a pull-off strength 30 times that of cylindrical pillars (del Campo et al., 2007). Spatular tips provided an intermediate degree of adhesion, while concave tips and spherical tips were slightly better than flat cylindrical pillars. Spuskanyuk et al. (2008) hypothesized that part of this larger adhesive force is due to the fact that mushroom-shaped tips are less adversely affected by edge defects than flat or cylindrical pillars (Spuskanyuk et al., 2008). Further, stress concentrations are reduced at the contact interface (Li et al., 2019). Several other papers (Aksak et al., 2014; Balijepalli et al, 2016, 2017; Fleck et al., 2017; Gorb et al., 2007a) have examined the mushroom shape both experimentally and numerically and concluded that it is one of the best pillar designs for adhesion. Fleck et al. (2017) and Balijepalli et al. (2017,2016) considered fibril detachment as a crack propagating along the pillar-substrate interface and found that mushroom-shaped pillars reduce the corner stress intensity of the contact zone, thus reducing the likelihood of detachment (Fleck et al., 2017) (Balijepalli et al, 2016, 2017). Review papers on the subject (Boesel et al., 2010; Li et al., 2019; Russell et al., 2019; Wang et al., 2020b; Zhou et al., 2013a) have also noted several other benefits of the mushroom shape, including improved adhesion enhancement via contact splitting and increased crack trapping compared to flat cylinders.

Figure 17D shows a three-layered hierarchical arrangement of polyurethane fibers with mushroom-shaped tips. This arrangement was manufactured with soft-lithography and capillary molding. Fiber aspect ratio, fiber radius, hierarchical branching arrangements, and material selection are all important factors as well. Figure 17 shows some of the tradeoffs that come with different manufacturing processes. While the polyurethane tips in Figure 17D have controlled tip geometry, their aspect ratio and fiber density are much lower than those of the carbon nanotube tipped design in Figure 17B. Neither of these designs was able to incorporate the tilted structure shown in Figure 17C. The gecko pad's ability to optimize all of these different parameters simultaneously provides just another example of why natural keratin can be so impressive relative to manufactured materials and how there is so much for engineers to learn from nature.

The applications for gecko-inspired, dry, reversible adhesives are seemingly endless. One of the most popular uses of this emerging technology is soft robotics (Li et al., 2016). The reversible dry adhesion is ideal for (unsurprisingly) climbing and gripping. It has been utilized for numerous commercial devices (like Onrobot's soft gripper and GECOMER's pick-and-place robotic systems), as well as countless academic pursuits (Arzt et al., 2021; Asbeck et al., 2009; Dadkhah et al., 2016; Estrada et al., 2016; Hawkes et al, 2015a, 2015b; Henrey et al., 2014; Jiang et al, 2015, 2017; Kalouche et al., 2014; Ko et al., 2017; Purtov et al., 2015; Seo and Sitti, 2013; Song and Sitti, 2014; Zhou et al., 2013b). Furthermore, several products utilizing adhesive materials based on the gecko pad are now commercially available from Geckskin, nanoGriptech, and Gottlieb Binder GmbH.

By way of their tunable and hierarchical structure, keratinous materials have evolved diverse methods to achieve reversible adhesion. In the feather, this is accomplished through the mechanical interlocking of hook-shaped barbs and barbules, while the gecko pad adheres to surfaces with van der Waals forces generated by its branched setal arrangement. These features have been translated to scaled up to macroscopic engineered systems (as in the feather-inspired 3D prints) and biomimetic nano- and microscale structures (for the gecko setae).

### Lightweight structures

In engineering applications, sandwich structures are used for their ultra-lightweight, energy absorption capabilities, and comparable mechanical strength relative to bulk materials. Sandwich structures can be tailored by controlling the properties of the face (outer cortex) and core (foamy center) and their geometry. Typically, sandwich structures are constructed with a high modulus face and a low modulus core to achieve a lightweight yet stiff material with rectangular cross-sections. Sandwich structures are not limited to engineered materials and are found in abundance in keratin-based systems, including beaks (Seki et al., 2006), feathers (Liu et al., 2015a; Sullivan et al., 2017b), quills (Yang et al., 2013), baleen (Wang et al., 2019), and spines. Unlike engineered materials, the faces and core of biological materials are frequently made of the same material but occur in distinct phases: the face being more compact while the core is more porous. Here, we will review how the lightweight yet mechanically robust, keratin-based sandwich structures implemented in porcupine quills and hedgehog spines serve as the basis of lightweight bioinspired designs. While sandwich structures are not limited to keratinous materials, this review highlights the structural and functional diversity found in keratin systems that lend themselves to developing bioinspired structures.

The porcupine quill is composed of α-keratin and is a lightweight yet buckling-resistant structure that undergoes significant compressive and flexural loads during its service as a protective mechanism. The sandwich structure of the porcupine quill consists of a thin-walled cylindrical cortex enclosing a closed-cell foam. Some porcupine quills contain an additional structural element that reinforces the foamy center, which is referred to as a stiffener. The stiffeners have been found to increase the compressive strength and buckling resistance of porcupine quills (Yang et al., 2013). Inspired by the stiffeners present in the porcupine quill, Tee et al. (2021) developed several 3D-printed cylinders with varying infill structures from uniform to non-uniform designs to mimic the radial structures found in the porcupine quill. However, mechanical testing was not performed, and little information is known on the degree of reinforcement the stiffeners provide and how their structure can be tailored (Tee et al., 2021).

Hedgehog spines are similarly structured to porcupine quills and contain reinforcing stiffeners, further classified as longitudinal stringers and transverse plates (Vincent, 2002; Vincent and Owers, 1986). Despite their structural similarities, porcupine and hedgehog spines serve different functions. Hedgehog spines are adhered to within the skin and are primarily used as shock absorbers upon falling from great heights, while porcupine quills can readily detach from the body and serve as a defensive mechanism. Due to their high stiffness and capabilities for impact resistance, hedgehog spines are a suitable inspiration for developing lightweight yet mechanically robust bioinspired designs.

Drol et al. (2019), using X-ray microcomputed tomography, were able to capture the key internal structural design elements found in hedgehog spines, which were then used to create computational model abstractions in ABAQUS and compared to analytical models to better understand the role that stringers and plates play in the spine's flexural performance. Ten models with increasing complexity were generated. The most basic level is a simple hollow cylinder (level 1) and builds up to most realistically represent the spine (level 10) with a complex arrangement of longitudinal stringers and periodically arranged branched transverse plates. The beam models were subjected to 3-point bending with a displacement-controlled boundary condition in which the bending stresses, the normalized bending stresses, and the Von Mises stress contours were quantified. The hollow tube, the simplest case, is reported to have the highest specific stiffness; however, the lack of stiffeners limits its ability to reduce buckling. The model with the next highest effective stiffness is model 10, the most complicated and representative model of the spine. Model 10 contains longitudinal stringers and branched transverse plates with the smallest spacing between the central plates and the longitudinal stringers and a more accurate curvature connection between the stringers instead of a blocked fillet. The build-in model 10 allows for removing material while maintaining stiffness, creating a lightweight yet stiff structure. The longitudinal stringers aid in increasing the bending stiffness by localizing material further away from the central axis, which effectively increases the second area moment. The transverse plates provide reinforcement and help distribute the applied load evenly, minimizing buckling and localized failure. Furthermore, this study provides insight into how the structural organization of keratin-based materials, such as the hedgehog spines, can be directly translated to synthetic designs to develop tailored stiff and lightweight structures. This study's findings have even inspired the development of novel football helmet liners to help reduce traumatic brain injuries. This example illustrates how bioinspired designs stimulate innovation (Drol et al., 2019).

The feather shaft is another example of how keratin can be used to achieve a lightweight yet mechanically robust structure that is able to withstand aerodynamic loads during flight. This behavior is primarily attributed to the sandwich structure of the feather shaft. The feather shaft is composed of an outer shell of compact keratin that surrounds a medullary center made of foamy keratin. Liu et al. (2015a) investigated the hierarchical structure and mechanical properties of the peacock tail feather shaft under tension and compression. They determined that the presence of the foam center enhanced failure resistance by delaying splitting and buckling of the cortex shell and exhibits overall improved compressive stability (Liu et al., 2015a). While there has been a significant amount of work dedicated to understanding the structure and mechanical properties of the feather shaft, there have been limited attempts toward the development of bioinspired sandwich structures based on the feather shaft. We suggest that this is an area of study for future work.

Many keratinous materials manage to achieve good mechanical properties while limiting their mass. Often this is accomplished with a sandwich structure consisting of foam surrounded by a stiff exterior face. Since low density is a highly coveted trait in engineered materials, these natural keratinous systems have served as the basis for bioinspired designs aimed to capture high strength to weight ratios.

### Structural color

**Figure 18:**
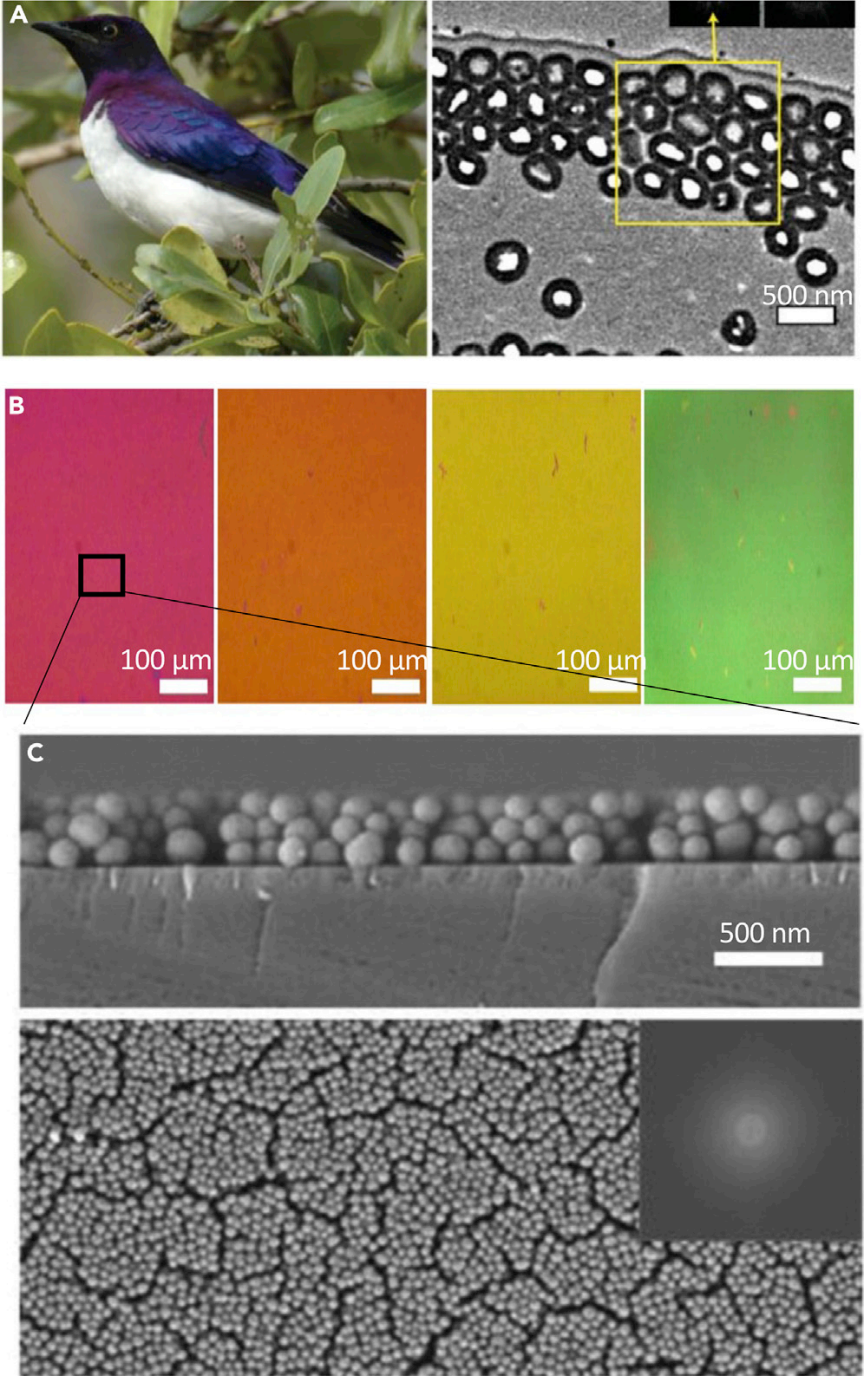
Structural color found in avian feathers and bioinspired analogs (A) Violet-backed starling and TEM micrograph of the multi-layered structure of hollo melanosomes and a thin film of keratin. (B) Structural color produced by SMNPs. (C) Micrograph detailing the arrangement of SMNPs as a thin film. Adapted with permission (Xiao et al., 2015). Copyright 2015, American Chemical Society.

Besides the outstanding mechanical, lightweight, and thermal properties of avian feathers, these keratinous materials are also known to display a diverse range of vibrant colors. This property is in part due to structural coloration, which arises from the interactions of light with a submicron array of varying morphologies which include multilayer structures (as seen in the iridescent throat patch of the hummingbird) (Gruson et al., 2019), two-dimensional photonic crystals (as seen in peacock and mallard feathers) (Freyer and Stavenga, 2020; Stavenga et al., 2017; Weiss and Kirchner, 2010; Zi et al., 2003), or spinodal-like channel structures (Parnell et al., 2015) (as seen in the Eurasian Jay *Garrulus glandarius*). These nanostructures self-assemble and can occur as a multi-layered structure of β-keratin and a pigment-based protein (e.g., melanin, carotenoids), as shown in Figure 18A. The combination of structural color from the sub-micron array of keratin and the absorption from the pigment is referred to as color mixing. β-keratin has a low refractive index (∼1.5), but when implemented in a multi-layer structure, it allows for high reflectance and vivid coloration (Burg and Parnell, 2018). The presence of pigments strongly contributes to the vibrant coloration due to their high refractive indices and broad absorption spanning the UV-visible range. Structural color in avian feathers can occur as iridescent or non-iridescent and is strongly dependent on the underlying structure and organization. Typically, long-range order is responsible for producing iridescence, while short-range order is non-iridescent (Noh et al., 2010). Thus, structural color in avian feathers is highly tunable and thus a desirable candidate for bioinspiration.

Despite the vast arrangement of keratin in combination with pigmentation and the subsequent multitude of colors with varying optical properties (iridescent vs. non-iridescent) found in bird feathers, there have been limited ventures at bioinspiration. The most prevalent study that draws inspiration from feathers is the development of structural color produced by self-assembly of synthetic melanin nanoparticles (SMNPs) inspired by the assembled melanosomes in avian feathers (Xiao et al., 2015). Xiao et al. (2015) used a vertical evaporation-based self-assembly method to develop thin films of SMNPs with a wide range of colors (red, orange, yellow, and green) (Figures 18B and 18C). The coloration produced is attributed to the thickness of the thin film which can be controlled by the concentration and evaporation rate. Additionally, the morphology of the SMNPs influences the packing, and, therefore, the film thickness and coloration produced. In avian feathers, there exists a diverse range of melanosome geometries from spherical to oblong and hollow to filled. These morphologies can additionally tune the coloration produced, which is an exciting avenue for future work. The SMNPs have a broad absorption spectrum (high absorption at short wavelength and low absorption at long wavelengths) and a relatively high refractive index (∼1.4–1.6 at 589 nm) which was found to be responsible for the enhanced color saturation and purity. In addition to the desirable optical properties, SMNPs are biodegradable and inherently biocompatible, making them suitable candidates for various applications (Xiao et al., 2015).

Structural coloration is not limited in nature to keratinous materials and is additionally found in chitin-based materials such as the morpho butterfly and the exoskeletons of beetles (Fu et al., 2016). These chitin-based systems have been extensively studied and have led to the development of numerous bioinspired structural colors (Chung et al., 2012; Steindorfer et al., 2012; Zhang and Chen, 2015). Despite their lack of prevalence in bioinspired structural colors, there are still many opportunities awaiting to be explored in the field of avian feathers. This review highlights the importance of keratinous structural colors found in avian feathers and the vast potentials for these systems to serve as bioinspired candidates.

### Hydrophobic surfaces

Hydrophobic surfaces are essential in both the engineering and the biological worlds. As such, researchers have been attracted to how living creatures can repel water by manipulating the contact angle of water droplets on their surfaces. Certain organisms, like ducks, excrete hydrophobic oils that can be spread on the surfaces of their feathers to repel water. Others, like the famous lotus flower, utilize nanoscale roughness to decrease the contact area of water droplets on their surface, with the two-fold benefit of keeping the organism dry while cleaning dirt and debris of the substrate as water droplets runoff. This phenomenon, dubbed the “lotus effect,” has been observed in several keratinous systems as well, which is particularly intriguing because keratin itself is very water absorbent. It should be noted that, in nature, several hydrophobic strategies are often utilized in tandem. The duck feather, for example, also has significant surface roughness, which helps to repel water along with the oil excreted by its uropygial gland, while the lotus leaf's nanostructure is covered by a thin, hydrophobic wax film that helps prevent water from penetrating the epidermis. Thus, researchers have attempted to imitate the surface features found on hydrophobic keratinous systems to create synthetic, water-repellent materials.

**Figure 19:**
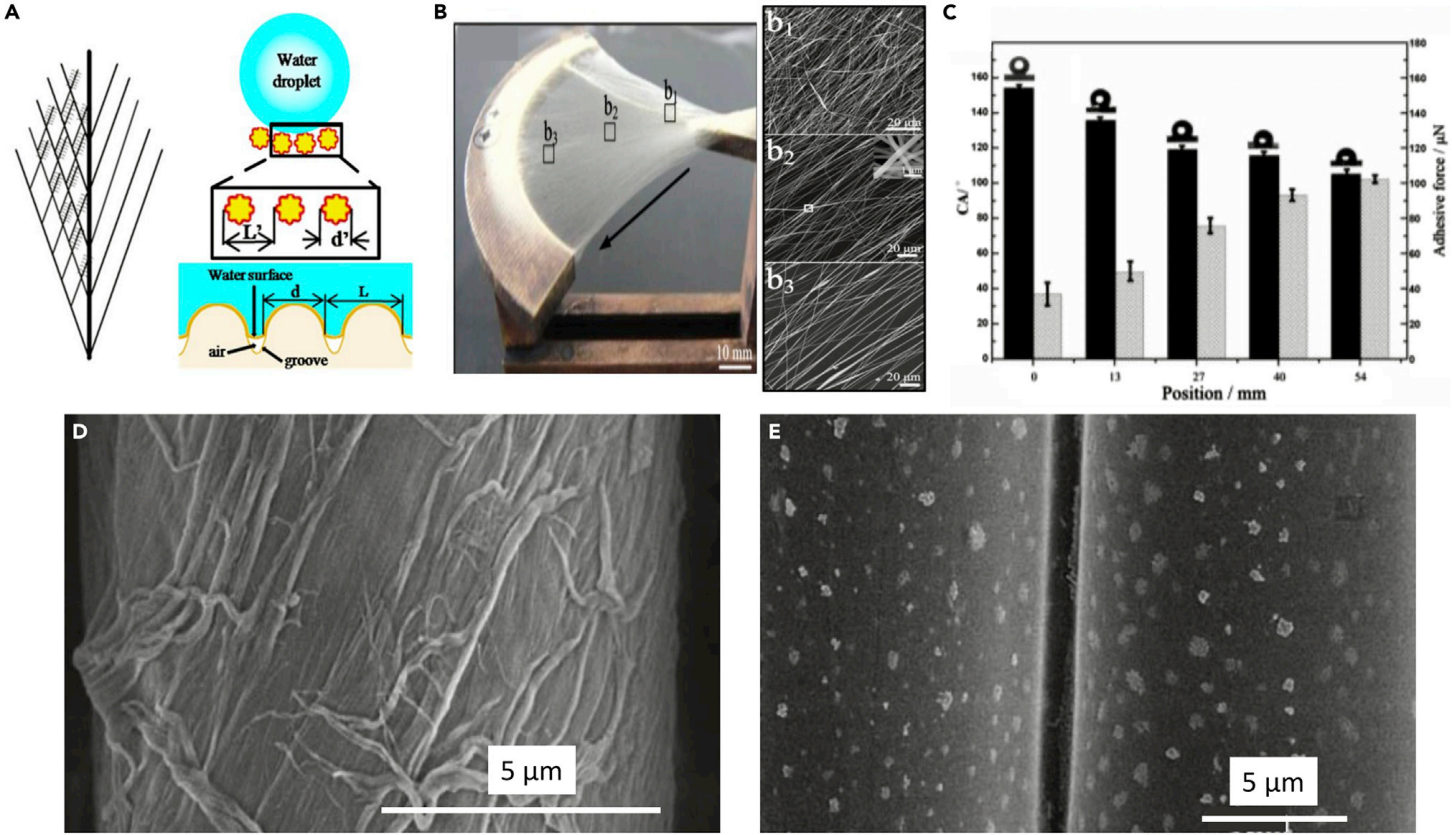
The multiscale surface roughness and fine nanoscaled grooves on feathers help them repel water (A) Schematic showing how the hamuli on penguin feathers trap air beneath water droplets creating an air cushion and minimizing the amount of material in contact with the water. (B) Bioinspired polyamide nanofiber membrane fabricated via asymmetric electrode electrospinning. (C) Chart of contact angle and adhesive force versus location on the polyamide membrane highlighting the effect of fiber density. Reproduced with permission (Wang et al., 2016d, 2016e). Copyright 2016, American Chemical Society (D) SEM image of cotton fiber with precipitated chitosan nanoribbons on the surface inspired by duck feathers. (E) SEM image of polyester fibers with precipitated chitosan “nanoflowers” on the surface. Reproduced with permission (Liu et al., 2008). Copyright 2008, IOP Publishing Ltd.

Penguin feathers have not only superior thermal insulating properties but also remarkable anti-icing properties. Despite spending a significant amount of time in freezing temperatures and swimming underwater, ice crystals are not typically observed on penguins' feathers. The secret to the ice-phobicity of penguin feathers is in its rough micro and nanostructure, which traps air in grooves, preventing supercooled water droplets from adhering and coalescing. A schematic of this water repulsion mechanism can be seen in Figure 19A. This trapped air is also postulated to provide a thermal barrier that reduces ice adhesion strength and heat transfer during icing. On the surface of the barbules and hamuli are grooves that are about 100 nanometers deep. These grooves are responsible for the surface roughness that creates the air pockets shown in Figure 19A (Wang et al., 2016d, 2016e).

Inspired by the penguin feather, Wang et al. (2016) used asymmetric electrode electrospinning to weave an anti-icing polyamide nanofiber membrane, as shown in Figure 19B (Wang et al., 2016d, 2016e). The radially arranged fibers mimic the barb tips' structure, while other fibers randomly overlap this arrangement, creating a regular 3D network similar to that found in the feather. The fibers are densely packed near the triangular electrode compared to the fibers near the curved electrode, as shown in Figure 19 b1, b2, and b3. This fiber arrangement creates a gradient in the chemical surface structure. In the region of densely packed fibers, the static contact angle of water droplets was ∼154° with a low adhesion force of ∼37 μN. In this region, droplets struggled to permeate the tightly bound membrane. The few droplets that were able to adhere to the fibers coalesced with other droplets and resulted in self-propelled jumping, i.e., the droplets fell off the membrane naturally before freezing. As the distance between the fibers increases, more droplets were able to penetrate the membrane. The static contact angle and adhesion force of the water droplets were measured at 105.1° and 102μN, respectively. Figure 19C shows the gradual change in these values through the membrane's radius. Droplet coalescence and jumping did not occur when the fibers' distance was greater than the diameter of droplets. After 3-4 hr at -5°C, some frost and ice were found on the less densely packed fibers but not in the densely packed fibrous network. This result shows that by tuning the density of the overlapping nanofiber network, anti-icing properties similar to those found in keratinous penguin feathers can be achieved.

Waterfowl, ducks in particular, are so famous for their anti-wetting capabilities that the phrase “like water off a duck's back” has worked its way into our everyday lexicon. Until recently, it was generally thought that this extraordinary hydrophobicity arose from the low surface energy of preening oil excreted from glands at the base of their tail and spread over the feathers. However, recent studies (Nishino et al., 1999) of preening oil on smooth surfaces have revealed that it is not that special after all and is less hydrophobic than several synthetic resins and oils. The feather's structure, coupled with preening oil, makes water run off of a duck's back so efficiently. Like penguin feathers, duck feathers have multiscale textures, with the same branched structure and micro-sized surface features covered with nanoscale grooves and protuberances. Liu et al. (2008) mimicked this structure by precipitating chitosan nanostructures on the surface of textile microfibers. They did so by dip-coating fibers in an acidic solution containing chitosan before placing them in an ammonia gas environment. The ammonia is absorbed by the film, making the solution basic and causing the cationic polyelectrolyte chitosan to precipitate in nanofeatures on the textile substrate's surface. On cotton fibers, the chitosan formed long ribbons (Figure 19D), while on polyester fibers, the chitosan shrank down to nanosized flower shapes (Figure 17E). The result is a hierarchical arrangement of surface irregularities where the fibers themselves compose the microscale roughness, while the chitosan precipitates form the nano roughness. Once the fibers are dried, they are treated with polysiloxane to lower the fibers' surface energy (similar to the preening oil found on natural duck feathers). With just the polysiloxane treatment, the contact angle of a water droplet was 118° for cotton and 100° for polyester. When combined with the chitosan surface roughness, these values rose to 152° and 148°, respectively, showing how the combination of a low surface energy film and surface roughness, similar to feathers, can lead to the development of superhydrophobic materials (Liu et al., 2008).

**Figure 20:**
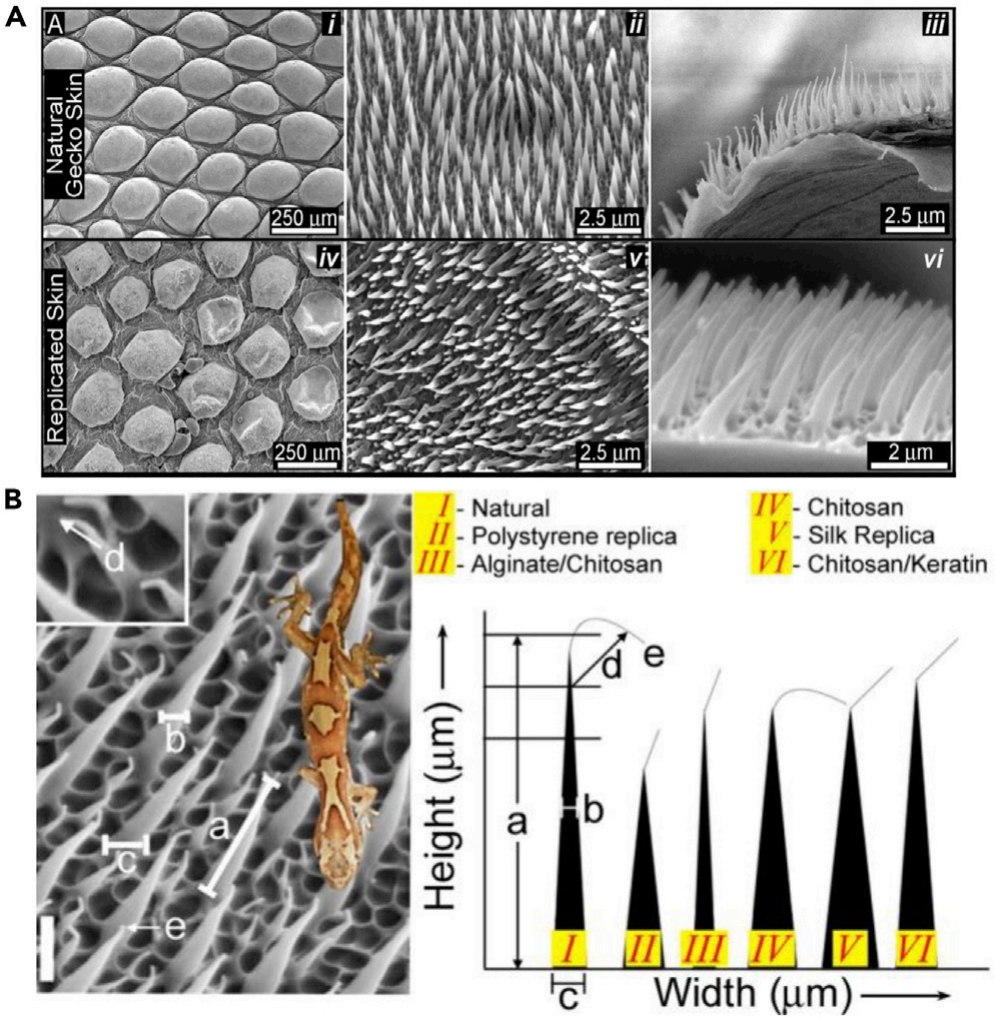
Much like the gecko pad, the outer layer of skin on the gecko has hydrophobic, self-cleaning properties due to its rough mesostructure, which researchers have attempted to replicate (A) SEM images of natural gecko skin (i-iii) alongside SEM images of biomimetic polystyrene replicas made via biotemplating (iv-vi). (B) Close-up SEM images of gecko spinules and the different measurements used to characterize them (left). Various biomimetic replicas, like the ones shown in A iv-vi, were prepared using several polymer solutions. The resultant spinule shapes are visualized (right) and compared to the natural spinules found on the gecko. Open access (Green et al., 2017). Copyright 2017, the authors.

The oberhautchen (thin outer layer) of many lizard and gecko skins is composed of β-keratin. Many geckos also have tiny keratin spinules upon this outer layer that serve to repel water and disrupt bacterial growth. Figure 20A (i-iii) shows images of the scales of *Strophurus williamsi*, a species of arboreal geckos found in Australia. Generally, these spinules are 0.5–4 μm long packed closely together with over 400 spinules per 10 μm^2^. These spinules are mounted upon scales that have a honeycombed-shaped basal layer composed of intersecting ribs. The static contact angle of water droplets was similar to that of feathers, ranging between 151 and 155°. Interestingly, gecko skin accomplishes such impressive superhydrophobicity from its spinule density rather than finer roughness structuring like the channels found on insect hairs or the hamuli in penguin feathers. These hairs not only prevent water from building up on the skin of the gecko but also allow the skin to clean itself, removing harmful bacteria and contaminants as droplets coalesce and run off the gecko with even the slightest tilt or perturbation (Watson et al., 2015).

Green et al. (2017) developed a benchtop biotemplating apparatus to fabricate synthetic replicas of gecko skin spinules with comparable hydrophobicity to emulate their antibacterial properties. To do so, negative molds were generated by coating shed gecko skin, which was adhered to a glass slide by a thin layer of water, with commercially available PVS. The water also served to inflate the spinules to mimic their natural state better. This negative mold was then used to fabricate gecko skin replicas from several different polymer solutions targeted toward various applications. These included a synthetic polystyrene solution and natural biopolymer solutions of chitosan, silk fibroin, fused bilayers of chitosan and alginate polysaccharides, and blended α-keratin hair extract (Green et al., 2017).

Each solution was successfully used to form a replica of the gecko skin nanostructure; several images of the natural shed gecko skin compared with the polystyrene replica are shown in Figure 20A (iv-vi). Some of the solutions were able to more closely mimic the gecko spinules' dimensions, as visualized in Figure 20B. The curing process had a significant effect on the ability of each solution to closely resemble the geometry of the natural gecko spinules. For example, the polystyrene solution hardens slowly due to organic solvent evaporation, which resulted in stiffer spinules with less curvature. The metrics for measuring curvature in the spinules are shown by images “d” and “e” in Figure 20B. The chitosan-based replicas, on the other hand, closely mirrored the curvature of the nano tip, as well as the thickness and height of the natural spinules. The biomimetic samples were only slightly less hydrophobic than the natural gecko skin obtaining a contact angle of about 134°. The synthetic spinule arrays also revealed notable anti-bacterial properties. Confocal microscopy showed that the spinules effectively disrupted bacterial cultures grown on the replicas removing as much as 95% of bacteria from the surface after water treatment. Vucko et al. (2008) developed a similar procedure using epoxy molds of live geckos to observe their oberhautchen without needing to kill and prepare them for SEM examination. This approach is highly applicable to other organisms and other research fields since it can be non-destructively performed on living creatures while generating finely detailed replicas for observation or functional use (Vucko et al., 2008).

Many keratinous materials that provide thermal insulation also protect organisms from getting wet because significant surface roughness benefits both areas. In the case of hydrophobicity, this roughness comes in many forms in nature, such as hamuli, nano grooves, or spinules, but all have the objective of reducing the area in contact with water droplets allowing them to run off the surface efficiently. Like research on thermal insulation and reversible adhesion, studies on bioinspired surface roughness to achieve anti-wetting properties have shown great success and are a promising research area for bioinspiration.

## Keratin as a material for engineered systems

So far, we have seen how keratinous structures provide beneficial properties that can be used to inspire engineered designs. However, keratin itself has often been utilized as a material for various applications due to its unique intrinsic properties. Over the past few decades, many researchers have explored how to connect different technologies such as materials science, applied health sciences, and engineering. This section will discuss possibilities to use keratin for applications in the: (i) biomedical, (ii) composite, and (iii) reversible material realms.

Historically, keratin was one of the first polymers used by humans before the plastics revolution in the 20^th^ century. Keratin extracted from tortoise shells has been used to craft fine components, like hairbrushes, for hundreds of years, while baleen from whales was famously used to make corsets (McKittrick et al., 2012; Wang et al., 2016b). Hair (typically human or horsehair) has also had versatile applications ranging from paintbrushes to the torsional springs used in ancient Greek and Roman artillery. Researchers have recently explored natural macromolecules as candidates to perform biochemical, mechanical, and structural roles due to their appealing properties.

Keratin can be extracted from various sources (typically wool, poultry feathers, or hair) using multiple different techniques. Common extraction methods include oxidative and reductive extraction, steam explosion extraction, or ionic liquids and eutectic solvents (Feroz et al., 2020; Shavandi et al., 2017). Studies involving oxidative technologies and reductive extraction were initially applied to animal horns and hooves but were also used to extract keratin from wool and human hair. Early studies on the properties of extracted keratin led to increased interest in exploring keratin for medical applications. Among the first innovations were keratin powders for cosmetics, fibers, composites, and coatings for drugs (Beyer, 1907; Dale, 1932; Goldsmith, 1909; Rouse and Van Dyke, 2010).

### Biomedical usage

Recently, there has been a significant increase in the number of biomedical studies related to using keratin-based biomaterials. This variety of applications includes biomedicine, natural polymer flocculants, bioelectronics, biolubricant formulations, and manufacturing bone scaffolds (Roy et al., 2015). Keratin is widely used in biomedical applications due to its biocompatibility, lack of immune reaction upon transplant, good cellular interaction, and biodegradability (Dickerson et al., 2013; Rouse and Van Dyke, 2010).

Asia has taken the lead in keratin biomaterials research since the first medical application of pyrolyzed human hair by a Chinese herbalist dates from the 16th century (Zhen, 2005). In the modern age, scaffolds, hydrogels, powders, films, and fibers have been prepared, starting with early studies by Japanese scientists (Ito et al., 1982; Noishiki et al., 1982) in 1982 on vascular graft production with hemostatic properties. Researchers have also shown that keratin can be effectively used for peripheral nerve regeneration, drug delivery, hydrogel formation, and films that promote wound healing (Rouse and Van Dyke, 2010). For medical applications, keratin has shown interesting characteristics, but its potential has not yet been fully explored. For example, areas such as wound healing, bone regeneration, peripheral nerve repair, antimicrobial activity, hemostasis, and cell adhesion of amino acid sequences (due to the Arg-Gly-Asp and Leu-Asp-Val binding motifs) have led to increasing interest in keratin for medical applications. Although keratin-based biomaterials show wide promise, there can be significant costs associated with the extraction and processing of keratin and its post-processed mechanical characteristics. In 1983 and 1985, researchers from Japan and the UK, respectively, published papers speculating on the prospect of using keratin as the building block for new biomaterials (Jarman and Light, 1985; Various Authors, 1993).

Also, keratin biomaterials derived from wool and human hair have been shown to possess cell-binding motifs, such as leucine-aspartic acid-valine (LDV) and glutamic acid-aspartic acid-serine (EDS) binding residues, which are capable of supporting cellular attachment. Together, these properties create a favorable three-dimensional matrix that allows for cellular infiltration, attachment, and proliferation. Thus, the conservation of biological activity within regenerated keratin biomaterials could prove advantageous for controlling specific biological functions in various tissue engineering applications (Rouse and Van Dyke, 2010).

Reconstituted biopolymers often suffer from inferior mechanical properties, which can pose a challenge for processing and limit applications. This is especially true for biomaterials made from extracted keratin fibers, despite the stellar mechanical properties found in natural keratinous materials. Thus, many studies have targeted keratin films, focusing on the physical strength and flexibility of the films while maintaining their excellent biological activity (Rouse and Van Dyke, 2010). The addition of other biopolymers such as chitosan or silk-fibroin improves the mechanical properties of keratin. The chitosan-keratin films also had beneficial anti-microbial properties and proved to be suitable substrates for cell cultures (Lin et al, 2017, 2018; Tanabe et al., 2002). For silk-fibroin and keratin films, studies have shown that the two molecules interact synergistically and provide unique properties not found in pure keratin or silk-fibroin films. For example, the polarity of keratin's amino acids causes silk-fibroin to rearrange from a random-coil to β-sheet configuration (Lee and Ha, 1999; Vu et al., 2016). As a result of these unique interactions, the combined film is more biocompatible (Lee, 2001; Lee et al., 1998) and biodegradable (Vasconcelos et al., 2008) than its constituents.

Keratin has also been explored as a raw material for cell scaffolds and shows significant promise due to its ability to self-assemble into complex 3D shapes. A host of fabrication techniques from electrospinning (Wang et al., 2016d, 2016e), wet spinning (Yue et al., 2018), photomask micropatterning (Yue et al., 2018), and compression molding/particulate leaching (Katoh et al., 2004b) to freeze casting of aqueous keratin solutions (Lin et al., 2019; Tachibana et al., 2002) have been used to create keratin scaffolds. These scaffolds have many advantages, including a stable homogeneous, interconnected, porous structure (Lin et al., 2019; Tachibana et al., 2002), free cysteine residues that can be used to bind bioactive substances to the scaffold surface (Kurimoto et al., 2003; Tachibana et al., 2005), and resorbability (Peplow and Dias, 2004) that make it a suitable material for tissue engineering and drug delivery (Lin et al, 2017, 2018, 2019; Srinivasan et al., 2010; Verma et al., 2008). These properties have also led to studies on keratin-based biomaterials for wound (Konop et al., 2017; Lin et al., 2018; Than et al., 2012; Wang et al., 2016d, 2016e) and burn dressings (Poranki et al., 2014).

Composite films of keratin and synthetic polymers have also been fabricated to create films with even better mechanical properties. For example, poly (diallyl dimethylammonium chloride) and poly (acrylic acid) were blended with keratin extracted from wool to fabricate thick films based on the principle of poly ionic complexation. This was accomplished using a layer-by-layer self-assembly method (Ducheyne et al., 2017; Katoh et al., 2004a; Sionkowska et al., 2010). Keratin blends with poly(ethylene oxide) have also been explored for usage as scaffolds for cell growth, wound dressings, and drug delivery membranes, while keratin mixed with polyamide 6 has been envisaged as a practical material for biomedical devices, active water filtration, and textile fibers (Zoccola et al., 2007).

Keratin's emerging role as a medical biomaterial revolves around many of the same aspects that make it a successful biological material. Its tunable properties and architecture make it viable for numerous different applications, while its abundance and natural origin make it appealing to researchers as an economical, sustainable, and biocompatible material. However, it is limited by the mechanical weakness of reconstituted keratin and the lack of cheap and scalable extraction techniques (Shavandi et al., 2017).

### Composites

Composite materials have steadily grown in popularity over the past decades due to their lightweight yet mechanically robust properties. However, these synthetic materials are traditionally produced from petroleum-based plastics, which are increasingly expensive and environmentally harmful. Many researchers aim to tackle this problem with biodegradable, renewably sourced composites made of biopolymer matrixes and natural fibers. Knowledge of the properties of available biodegradable polymers and natural fibers is essential for manufacturing a biodegradable composite (Shrivastava and Dondapati, 2021).

Polymers reinforced with natural fibers, commonly named “bio-composites,” have started to be used industrially in the automotive and building sectors as well as the consumer goods industry. Green composites are a specific class of bio-composites where a bio-based polymer matrix such as a biodegradable polyurethane is reinforced by natural fibers such as keratin (García et al., 2008; Quirino et al., 2014; Zia et al., 2008). Väisänen et al. (2016) describe natural fiber-polymer composites (NFPCs) as renewable and sustainable materials since they are composed of natural fibers embedded in a polymer matrix which may also be of biological origin (e.g., PLA) (Väisänen et al., 2016).

Conzatti et al. (2013) reported on the valorization of keratin-based wastes, made of unserviceable poor quality raw wools from farm breeding, fiber byproducts from textile processing, and horns, nails, hair, and feathers from butchery. Zoccola et al. (2009) estimated that keratin wastes from breeding, butchery, and textile industry, made up of wool, hair, feathers, beaks, hooves, horns, and nails, have been estimated worldwide to be more than 5,000,000 tons/year (Zoccola et al., 2009). With an increasing demand for sustainable materials, these protein byproducts are beginning to be regarded as renewable resources worthy of better exploitation (Conzatti et al., 2013).

Extracted keratin has also gained popularity as a component for composite production as both a filler material and a fiber reinforcement. This interest is primarily driven by keratin's availability and environmental benefits (biodegradable, renewable, leftovers from other products) on top of their beneficial properties.

Donato et al. (Donato and Mija, 2019) discussed the manufacturing of keratin-based composites with different polymers in detail. To form efficient keratin-polymer composites, it is essential to have good adhesion between the fiber and polymer matrix. Since keratin fibers have numerous hydrophilic surfaces, this can lead to weak mechanical properties of the overall composite material. As a result, coupling agents are sometimes required to boost interfacial adhesion. For example, Song et al. (2017) used functionalized cellulose nanocrystals to crosslink keratin fiber while also serving as reinforcement. This interfacial treatment resulted in marked improvements in tensile strength, elongation to failure, and toughness of such a composite. Further, the incorporation of cellulose nanocrystals reduced the keratin's water sensitivity which is a barrier for many in vivo applications (Song et al., 2017). More approaches to coupling agents and keratin-polymer composites are discussed in detail by Shavandi and Ali (Shavandi and Ali, 2019).

As attractive as synthetic polymers are, their use is coming under scrutiny due to the realization that petroleum reserves are finite and that oil prices are likely to rise steadily over the next few decades. Furthermore, with global environmental awareness at an all-time high, synthetic polymers have lost some of their luster. The synthetic fiber industry as it currently exists will ultimately decline and be replaced by an industry based on renewable feedstocks (Fudge et al., 2010). Recent works on the mechanical properties of fibers isolated from hagfish slime suggest that these unique fibers may one day be replicated in a way that is environmentally sustainable and economically viable. These “slime threads” consist of bundles of 10 nm protein nanofibers known as IFs, which form part of the cytoskeleton in most animal cells (Koch et al, 1994, 1995). Keten et al. (2010) explored the nanoconfinement of β-sheet crystals in silk as a means to control stiffness, strength, and toughness. This study highlighted another feature that makes β-sheet crystals an attractive model: they self-assemble from soluble precursors into 10 nm filaments in aqueous buffers (Keten et al., 2010). The key to the high strength and toughness of spider silk and hagfish threads are the β-sheet crystallites that simultaneously crosslink the protein molecules and arrange them into a structure in which “sacrificial bonds” increase the energy required to break the material (Keten et al., 2010; Koch et al., 1994; Mostaert and Jarvis, 2007).

Pourjavaheri et al. (2018) developed a bio-composite from chicken feather waste and thermoplastic polyurethane. This composite material was fabricated via solvent-casting evaporation at eight different compositions. The thermo-mechanical properties of the composites were assessed using thermogravimetry, dynamic mechanical analysis, and stress-strain measurements with hysteresis loops. The results showed that keratin derived from a current waste product from the poultry industry could effectively and cheaply provide the thermo-mechanical properties required of composite materials (Pourjavaheri et al., 2018). Similarly, Tran and Mututuvari (2016) developed composite materials made from keratin, cellulose, and chitosan combinations. They found that adding cellulose and chitosan improved the mechanical and thermal stability of the overall material but hindered the reformation of α-helices. Instead, when combined with these biopolymers, the keratin preferred the extended β-sheet morphology or amorphous configurations (Tran and Mututuvari, 2016).

### Reversible materials

Another attractive characteristic of keratin as raw material is its mechanical reversibility. This reversibility can be found in keratin due to the transition from α-keratin helices to β-keratin sheets. This transition has been observed as a result of stress along the longitudinal axis of the α-helix, as well as heat absorption or from a combination of the two (Chapman, 1969a; Haly and Snaith, 1970; Hearle, 2000; Mason, 1965; Miserez et al., 2009). Recently, Cera et al. (2021) have captured this reversible process using hydration as a trigger to fabricate 3D-printed, hierarchical shape-memory materials out of keratin extracted from animal hairs. Impressively, this material had a tensile strength and Young's modulus orders of magnitude higher than conventional water-triggered shape-memory materials (Cera et al., 2021).

**Figure 21:**
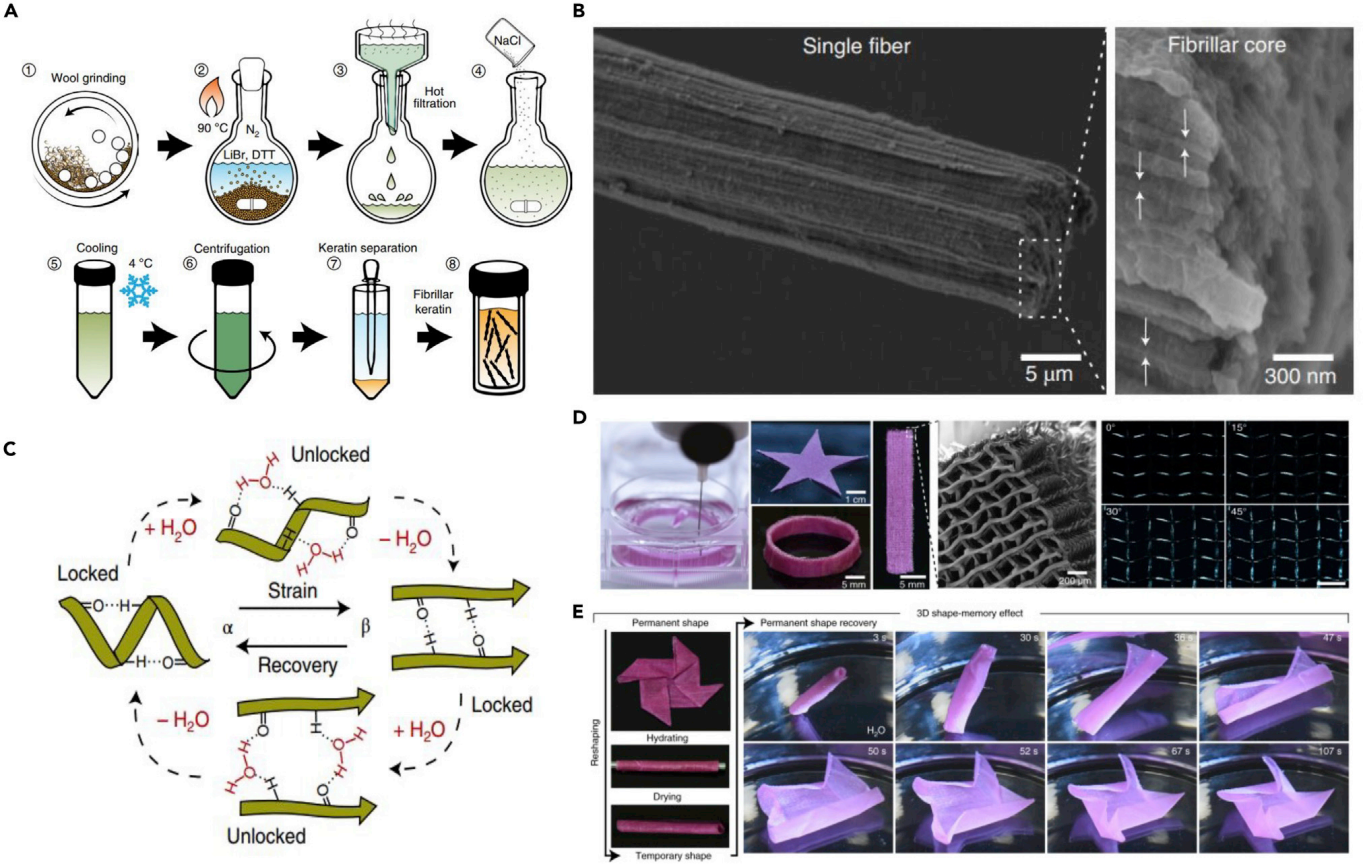
Advances in 3D printing technology have recently made printing different biological materials more feasible Cera et al. have utilized these advances to fabricate hydration-induced shape-memory components out of keratin. (A) The keratin extraction process used to obtain printable, fibrillar keratin (Cera et al., 2021). (B) To obtain aligned fibrils, keratin fibers were fabricated using traditional wet-spinning. The resultant hierarchical structure is visualized here. (C) Schematic of the atomic scale process for using water to lock and unlock the hydrogen bonds within α-helices or between the β-sheets. This mechanism endues the material with shape recovery properties. (D) Images of the keratin printing process and final products (left); SEM image of the fine detail that can be obtained; birefringence images showing the alignment of the keratin fibers in the woven structure. (E) Series of still images of the hydration-induced shape recovery of the printed samples composed of keratin, showing the prints returning to their initial form over a matter of seconds when submerged in water. Reproduced with permission (Cera et al., 2021). Copyright 2020, the authors.

Fibrillar keratin was extracted from ground Angora wool using LiBr to induce a solid-liquid phase transition of the crystalline keratin and DTT to cleave the disulfide bonds in the hair matrix at 90 C^o^. The product was then filtered, cooled, centrifuged, and separated to obtain concentrated fibrillar keratin quantities. This extraction process is shown in Figure 21A. When subject to shear stress and spatial constraint, the extracted keratin protofibrils self-organized into a nematic crystal phase. Adding NaH_2_PO_4_ to the extracted keratin allows for tighter control of the nematic phase by introducing a charge screening effect which causes the keratin fibrils to interact more. This process makes the crystalized proteins stiffen and pack closer together with more alignment. The result is a shear-thinning, viscous, keratinous solution that is ideal for extrusion processing and whose properties can be tuned via the NaH_2_PO_4_ concentration.

To maximize uncoiling when loaded and to improve tensile strength and strain-to-failure, α-helices were aligned using traditional wet-spinning. Spun fibers were exposed to hydrogen peroxide to restore the disulfide network of the keratin. Figure 21B shows that the keratinous fibers (∼10 micrometers in diameter) maintained a hierarchical structure, with a core composed of fibrils that are approximately 50 nm in width. When stretched in the wet state, the α-helices unwind into β-keratin sheets. As the fibers dry while under a constant load, hydrogen bonds begin to form between the β-sheets, fixing them in place and making them metastable. In fact, when stretched to 80% strain and held in place for 10 min at room temperature, the fibers only shrunk back to 77% strain, showing the efficacy of these hydrogen bonds for locking the keratinous fiber into its new fixed shape. Upon rehydration, the hydrogen bonds are disrupted, and the fiber can return to its original shape. This process is visualized in Figure 21C.

Owing to the shear-thinning properties of the keratinous solution, small diameter extrusion needles can be used to print different geometries with textural features on a scale of 50 micrometers. The keratinous material was printed into a hydrogel which served as support, as well as the coagulation bath. The keratin protofibrils aligned themselves along the print pathway, allowing finer control of the material's shape memory properties. Figure 21D shows the 3D printing process to fabricate a flat star, ring, and flat strip. The middle image shows an SEM of the fine details that could be produced. The images on the right are birefringence images that show the common alignment of the keratin protofibrils. Once the keratinous material has been printed, it can be further manipulated into new shapes before the disulfide network is reformed by exposing the print to hydrogen peroxide. Figure 21E shows a square print that was folded into an origami star shape before the disulfide network was reformed. Once the star shape is set with the hydrogen peroxide, water can be used to trigger shape recovery even when it has been deformed into a tube. In this case, it takes less than 2 min for the tube to recognizably transform back into the star origami arrangement, as seen in Figure 21E (Cera et al., 2021).

## Conclusions

This review aims to establish a link between keratin as a fibrous biopolymer and as a material of engineering interest due to its wide-ranging functionality. Keratin fills many different niches in nature due to its inherent properties and its geometric tailorability on multiple length scales derived from its self-assembled hierarchical structure. We established the importance of each of these aspects by exploring keratin as a source of design inspiration alongside the keratin as a raw material for engineered systems.

Keratinous systems have been used to inspire materials with mechanical, thermal, reversible adhesive, lightweight, structural color, and hydrophobic characteristics. These bioinspired designs have not only been used to understand the success of biological materials better but have served also as a creative platform for researchers to extend natural design ideas beyond the limitations of nature, laying the groundwork for the next generation of functional materials. Keratin also has been used as filler or reinforcement in composites with an eye toward environmentally sustainable production and specific biomedical applications. Keratin's prolificity in the industrial world in wool and feathers alongside its beneficial material properties makes it a desirable constituent for expensive components like biomedical materials or fiber-reinforced composites.

## Future directions

Keratin has a lot to offer to the scientific and engineering communities, but several obstacles need to be overcome to convert its propitious potential into reality. Here, we suggest several future directions to maximize the impact of keratinous materials on the engineering and scientific communities:

• Material selection
  ○ As discussed in Sections 1 and 3, 1 and 3, keratin has a hierarchical structure that allows for tailorable material properties. When manufacturing bioinspired components, it can be challenging to find a material that matches the properties (i.e., Young's Modulus, strength, toughness, viscoelasticity, conductivity, density, and others) of natural keratin. This can make translations of natural keratinous designs to synthetic systems challenging. Recent developments in the 3D printing of keratin (Cera et al., 2021) have the potential to eliminate this issue by allowing bioinspired designs to be printed using keratin.
• Hierarchical structure
  ○ As shown in Figures 1 and 2, keratin has an inimitable hierarchical structure that plays an important role in its extensive functionality, i.e., atomic-scale hydrogen bonds in the amino acids make keratin's properties highly tunable via moisture alongside the nanoscale α-helices, which allow for a phase transition at 20% strain while mesoscale features like lamellae, spinules, or spatulae, toughen, repel water, or adhere to surfaces, respectively. Engineers have struggled to replicate the multiscale ordered arrangements found in keratinous systems that help them be so multifunctional, and this remains a major challenge for the field going forward.
• Exploration of additional keratinous materials in nature
  ○ While there has been significant research on various keratinous systems, there are other keratinous materials that have not yet been studied, particularly amongst reptiles and birds. Much of the research on keratin has revolved around its role in wool, hair, or human skin, which all possess the α-keratin. However, much less is known about β-keratin. Further, each keratinous system bears its unique structure optimized for its role in an organism. Exploring more keratinous systems will continue to reveal new design motifs and inspiration for engineered materials.
• More bioinspired designs
  ○ Similarly, some keratinous systems have been explored, but few attempts have been made to replicate their structure in synthetic materials. These include pangolin scales, butterfly cocoons, nails, talons, claws, and beaks, amongst others.
• Multifunctional bioinspired designs
  ○ Harrington et al. (2016) eloquently state: “in the case of biological materials, a battery of selective pressures encountered over the evolutionary history of the organism influence the final product,” and as such biological materials are always multifunctional (Harrington et al., 2016). However, engineers often replicate these materials with a singular objective in mind, ignoring the tremendous benefits of a multifunctional material. An exception is the gecko pad, where researchers have perused its reversible dry adhesion, self-cleaning capabilities, and toughness (Boesel et al., 2010; Li et al., 2019). Taking a multifunctional approach to each bioinspired design could help to develop superior materials that can be used for numerous applications at once.
• Focus on different length scales
  ○ A vast majority of the work on bioinspired keratinous materials has been done at the macro-, meso-, or micro-scale and is often scaled up for fabrication. An increased focus on generating these structures at their natural length scale could help recapture the original material's properties. Similarly, a broader thrust in exploring the nanoscale behavior of keratin could help develop hierarchical materials or unlock further functional mechanisms that larger-scale experiments have not revealed.
• Numerical and analytical modeling
  ○ Modeling is a beneficial way to understand the structure-property relationships, particularly for a complex biopolymer like keratin. Improved models would help to understand better the hierarchical synergies in keratin and which design parameters are most important for different functionalities.

Listed above are just some of the possibilities for future work on keratin as an engineering material. However, this list is not necessarily specific to keratin. Many other biopolymers like collagen, elastin, and chitin have similar wide-ranging usages in nature. Uncovering what niches each of these biopolymers can fill, how they succeed in so many different environments, and using them in engineered materials will provide a wealth of knowledge to the engineering community. All of this comes with the added benefit of biopolymers being renewable resources. With so many different utilities, understanding and replicating keratin-like structures has the potential to touch every corner of society.

### Limitations of the study

This study is not a comprehensive review of all applications of keratin, particularly in the biomedical field. Further, certain popular research topics discussed above, such as gecko pads, are not reviewed comprehensively. For a deeper understanding of these areas, the authors refer readers to the cited review articles.
